# Truth and Bias, Left and Right: Testing Ideological Asymmetries with a Realistic News Supply

**DOI:** 10.1093/poq/nfad013

**Published:** 2023-04-29

**Authors:** Bernhard Clemm von Hohenberg

**Affiliations:** Research Associate, Department of Computational Social Sciences, GESIS Leibniz Institute for the Social Sciences, Cologne, Germany

## Abstract

The debate around “fake news” has raised the question of whether liberals and conservatives differ, first, in their ability to discern true from false information, and second, in their tendency to give more credit to information that is ideologically congruent. Typical designs to measure these asymmetries select, often arbitrarily, a small set of news items as experimental stimuli without clear reference to a “population of information.” This pre-registered study takes an alternative approach by, first, conceptualizing estimands in relation to all political news. Second, to represent this target population, it uses a set of 80 randomly sampled items from a large collection of articles from Google News and three fact-checking sites. In a subsequent survey, a quota sample of US participants (n = 1,393) indicate whether they believe the news items to be true. Conservatives are less truth-discerning than liberals, but also less affected by the congruence of news.

##  

Behavioral and psychological differences between liberals and conservatives have been of interest to political science for decades (Adorno et al. 1950; Rokeach 1960). A large variety of studies have examined—and led to controversies about—whether left and right differ on behaviors and traits such as political tolerance (Sniderman et al. 1989; Crawford and Pilanski 2014), cognitive rigidity (Jost et al. 2003; Kahan 2016), knowledge and overconfidence (Jerit and Barabas 2012; Harris and Van Bavel 2021), and trust (Hetherington and Rudolph 2016; Morisi, Jost, and Singh 2019).

With the debate on “fake news” around the US elections of 2016 (Lazer et al. 2018), the question of whether liberals and conservatives differ in how they process the news has seen particular attention. It can be made more specific in two closely related ways: First, do liberals and conservatives differ in truth discernment, that is, the ability to tell true from false information? Second, since news can be more or less in line with certain attitudes or values: Do liberals or conservatives have a greater tendency for bias, that is, the tendency to believe information depending on whether it is ideologically congruent?

Several recent studies address these questions (e.g., Allcott and Gentzkow 2017; Swire et al. 2017; Ross, Rand, and Pennycook 2019; Pennycook, et al. 2020; Guay and Johnston 2022), but the evidence is mixed (see next section). Some find one side to be more truth-discerning or biased, while others yield no asymmetries. A recent meta study of over 50 experiments concluded that “bias is bipartisan” (Ditto et al. 2019), although this conclusion is debated (Baron and Jost 2018).

The design used in many studies requires confronting subjects with some previously selected news items. As I have suggested in a recent review, the selection or construction of informational stimuli is likely to influence results about asymmetries (Clemm von Hohenberg 2020). Unless studies clearly define a target population of information and ensure a valid representation of this population, estimates may be difficult to interpret and lacking robustness, as I illustrate below by reanalyzing data by Pennycook and Rand (2019).

I set out to test ideological asymmetries in truth discernment and bias with an alternative design. First, I conceptualize the target population of information, namely the entirety of political news, whether true or false. In a pre-registered procedure (http://egap.org/registration/6720), I then build two collections meant to approximate this target population, namely articles aggregated by Google News, and news reviewed by three fact-checking organizations, both for a time period of nine months in 2019 and 2020. I then randomly sample 80 news items from these two collections, which are then checked for truth and rated in terms of ideological valence in a pre-test.

Finally, I ask a quota sample of US adults (n = 1,393) to indicate to what extent they believe the news items to be accurate. Results show that conservatives are less truth-discerning than liberals. But liberals show greater bias: both for true and for false information, they believe congruent information to be more accurate than incongruent information. However, in substantive terms, ideological asymmetries are modest. In an exploratory way, I further simulate how differences change once we account for the possibility of selective exposure.

## (A)Symmetries in Truth Discernment and Bias

Misinformation was a daunting by-product of the 2016 US presidential campaign. Some observers suggested that by persuading conservatives and demobilizing liberals, it facilitated the election of Donald Trump (Grice 2017; Bovet and Makse 2019). Underlying such claims is the question of whether the ability to tell true from false information—*truth discernment*—is unequally distributed across the ideological spectrum.

Expectations on this question come from two angles: first, truth discernment might vary because of asymmetries in psychological characteristics. Scholars argue that conspiratorial thinking, closely linked to believing falsehoods, serves epistemic needs more common among conservatives (Douglas, Sutton, and Cichocka 2017; Jost et al. 2018; van der Linden et al. 2020). Similarly, if conservatives had lower levels of cognitive reflection than liberals (Iyer et al. 2012; but also see Kahan 2016), we might expect them to be less truth-discerning (Pennycook and Rand 2019).

A second line of reasoning is based on observations of the real-world misinformation supply, which appears to skew conservative: Guess, Nyhan, and Reifler (2018) found that over 90 percent of articles from “fake news” sites before the 2016 elections were pro-Trump. Sites peddling falsehoods were also more connected to the conservative media sphere (Benkler, Faris, and Roberts 2018) and received more social media interactions by conservatives (Guess, Nagler, and Tucker 2019). In principle, the supply of news responds to demand (Hamilton 2011; Munger and Phillips 2022) and misinformation entrepreneurs in particular are sensitive to people’s news interests (Gravino et al. 2022). Accordingly, a surplus of pro-conservative misinformation may indicate, simply, that conservatives are more gullible. This logic is illustrated by the story of Macedonian teenagers who converged to producing false stories catering to Trump supporters, rather than Bernie Sanders supporters, because it worked better (cf. Munger 2020).

Direct tests of ideological asymmetries in truth discernment, which typically confront subjects with a set of true and false news items—mostly without mirroring supply dynamics—yield mixed evidence: some find conservatives to be less truth-discerning (Allcott and Gentzkow 2017; Swire et al. 2017; Pennycook and Rand 2019; Ross, Rand, and Pennycook 2019), while others find no such asymmetry (Surma and Oliver 2018; Faragó, Kende, and Krekó 2020; Pennycook, McPhetres, et al. 2020). The inconclusive evidence leads me to ask: *Are conservatives or liberals better at discerning true from false information? (RQ1)*

News articles often have valence, that is, they are more or less in line with ideological predispositions. This raises the question of asymmetries in *bias*, which denotes the tendency to more easily believe information that is ideologically congruent, compared to incongruent information.^1^ One strand of research examines whether psychological characteristics that possibly drive bias are more common in one or the other group (e.g., Jost et al. 2003; Hibbing et al. 2014). For example, a “need for closure” might enhance bias by making the quest for consistent beliefs more urgent. Proponents of the “asymmetry hypothesis” argue that such traits are more prevalent among conservatives (Jost et al. 2003; Jost 2017). In contrast, the “symmetry hypothesis” holds that bias is rooted in socio-political identities and thus should be found across the spectrum (Ditto 2009; Graham et al. 2013). Similarly, according to the paradigm of motivated reasoning, people are *generally* motivated to resist information contrary to their belief system (Taber and Lodge 2006; Kahan 2013).

Again, the real-world news supply may provide a clue about asymmetries in biased processing. Partisan media on one or the other side may have a greater tendency to select news in line with an ideology (D’Alessio and Allen 2000; Shultziner and Stukalin 2021). By the mentioned logic of demand and supply, such selection may be driven by consumers preferring congruent over incongruent news, which could in turn indicate greater propensity to bias. Whether selective reporting is more common in the liberal or conservative media sphere is, however, contested (Hassell, Holbein, and Miles 2020).

Again, researchers directly testing asymmetries in bias typically select a set of news items, balanced in terms of ideological congruence, and then ask subjects to judge the items’ accuracy. Some of these studies do not find any asymmetry (Allcott and Gentzkow 2017; Faragó, Kende, and Krekó 2020; Pennycook, Bear, et al. 2020), while some do (Pennycook and Rand 2020). Another design randomly assigns subjects to one of two information stimuli with similar content but different valence, and tests for which ideological group the treatment effect is larger (e.g., Guay and Johnston 2022). A recent meta study of this design concludes that bias is symmetric (Ditto et al. 2019; debated in Baron and Jost 2018). Considering this mixed evidence, I ask: *Do conservatives or liberals show greater bias? (RQ2)*

Given that many papers study both truth discernment and bias, it is important to stress that the two are distinct phenomena. Although scholars theorize that whether we give more credit to true than false information depends on how congenial it is, the strength of one’s truth discernment does not necessarily predict one’s tendency for bias. For example, someone could be very able at distinguishing true from false news, but give much more credit to congruent over incongruent information; or, in contrast, not be very truth-discerning, but also not be overly impressed by the ideological congeniality of news.

Some studies suggest that people are better at discerning truth from falsehood when information is congruent (Pennycook, Cannon, and Rand 2018; Pennycook et al. 2019; Pennycook and Rand 2019). But there is no evidence on the flipside of the interaction—are people more biased for true or false news? Importantly, we do not know anything about ideological asymmetries in this interaction: *Is there an interaction between bias and truth discernment, and is it more pronounced for conservatives or liberals? (RQ3)*

## Defining the Population of Information

The inconclusive evidence on ideological asymmetries in information processing justifies a closer look at the typical methods. Most of the studies studying such asymmetries directly follow two basic steps: first, they make a selection of news stimuli; second, they ask people to rate these stimuli. One of the reasons for mixed results could be that the selection is not always comparable: researchers may set out to estimate whether conservatives and liberals show different abilities to discern true from false information—but what kind of information? Given the daily production of news content, the possible choices of such stimuli are infinite.

As I argued in a recent review (Clemm von Hohenberg 2020), when using such “stimulus selection” designs, researchers should strive for two things: first, to conceptually define the *population of information* they want to study; and second, to make an effort to achieve a valid representation of this population in their stimuli selection, ideally through random sampling. I will illustrate these points with a reanalysis of an existing study in the next subsection, before I conceptualize the population of information targeted in this study further below.

## Illustrating the Problem of News Item Selection

Pennycook and Rand (2019) study various aspects of truth discernment, one of which concerns the role of respondent ideology. It should be emphasized that this is only one of a rich array of questions their paper tackles. To date, the paper has hundreds of citations across the social sciences (e.g., Burger, Pfattheicher, and Jauch 2020; Guess, Nyhan, and Reifler 2020; Osmundsen et al. 2021). In Study 2, the researchers ask a sample of US subjects to rate the accuracy of 12 true and 12 false Facebook news headlines. Half of the headlines were pre-tested as favoring a Democrat, half of them a Republican worldview. More specifically, the pro-Democrat items were pre-tested as equally distant from the ideology midpoint as the pro-Republican items.

First, what is the population of information being studied? Although not stated explicitly, we could characterize it as the “population of news headlines that is balanced in terms of truth and ideological valence.” The benefit of balancing is that it allows us to compare psychological processes on a “level playing field.” Yet, the conceptual relevance of such a balanced population, and of ensuing estimates, is somewhat unclear, given that the headlines people encounter are arguably *not* balanced in terms of truth and valence.

Second, if we think that such a population of information is worth studying, can we be sure that the sample of news items made by the authors represents the population well? The problem is that there are many alternative headlines that *could* have been selected at the time of the study: there is no shortage of headlines on Facebook that are true, false, pro-Democrat, or pro-Republican. If estimates resulting from such alternative selections vary greatly and yield widely different asymmetries, then it is unclear what the right selection would have been.

**Figure 1.**
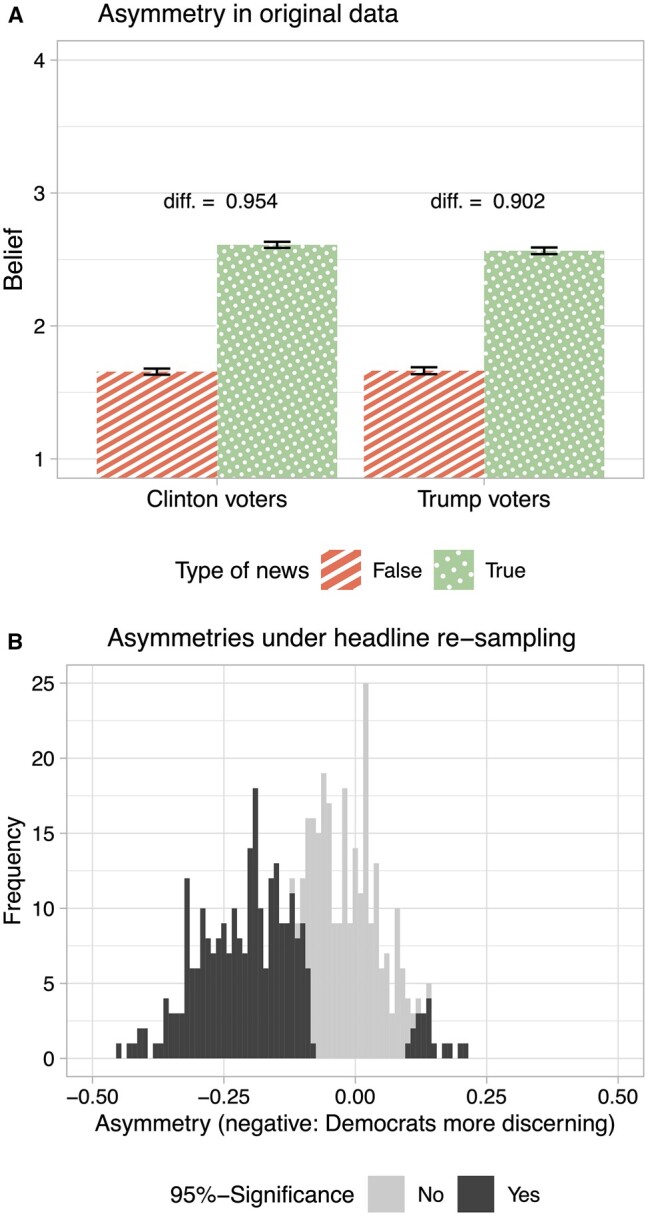
Reanalysis of Pennycook and Rand (2019) Study 2. Panel (A) plots the original results, according to which Republicans are slightly less truth-discerning. Panel (B) shows the distribution of asymmetries (average discernment of Republicans minus average discernment of Democrats) when randomly subsampling headlines 500 times, maintaining the balance of true/false and pro-Clinton/pro-Trump headlines. Negative values indicate greater discernment among Democrats. Subsamples for which two-way ANOVA is significant (*p* < .05) in dark.

The variance of estimates resulting from hypothetical alternative selections can be illustrated by a reanalysis of the original study. To do so, I first replicate the estimate of interest, which is the result of a two-way ANOVA of individual truth discernment scores (z-scored average belief in true news minus z-scored average belief in false news) on individual partisanship and ideological valence of the item. It reveals a significant effect of partisanship, which the authors summarize thus: “Clinton supporters were better able to discern fake from real news across the full range of items than Trump supporters” (Pennycook and Rand 2019, p. 7). The top panel of figure 1 replicates this original finding.

However, would that result hold against alternative selections? It is impossible to ask the same subjects about other headlines of the same time period. But imagine the authors had used 16 headlines instead of 24, maintaining the balance in terms of truth and ideological valence. This would seem no less justified conceptually. Hence, I reproduce the key result after repeated random sampling of 16 out of 24 headlines (i.e., four out of six headlines from each truth-by-valence category). Note that by reducing the number of stimuli this way, statistical power is unaffected, as the outcome measure is an individual-level average across items, so that re-sampling does not change the number of observations.

The right panel of figure 1 shows the distribution of truth discernment differences between Trump voters and Clinton voters for 500 random item subsamples. Positive values show subsamples in which Trump voters are more discerning, negative values those in which Clinton voters are more discerning. The proportion of differences that are significant are shown in dark. Overall, in only 49.2 percent of subsamples, Democrats show significantly better truth discernment. Thus, in half of all scenarios, the authors would have come to the conclusion that there was no ideological asymmetry, or even the reverse asymmetry.^2^

Of course, the authors got the correct results “on average”—however, we do not know to what extent this average is related to the target population. It should be emphasized that the authors had a reason for building their collection of stimuli: hypothesizing that ideological valence influences truth judgments, they controlled for this factor in their item selection. The resulting asymmetries can be interpreted as psychological differences with the information supply held constant this way. However, an alternative estimation strategy, which I pursue below, tries to represent an information supply that is likely *not* balanced this way. Instead of *balancing* stimuli on certain variables ex ante, one can *measure* these variables and compare asymmetries with and without a balancing *ex post* (see the results section).

## The Population(s) of Political News

How, then, might we conceptualize a theoretically interesting target population of information? Consider the following characterization of the real-world information environment. Every day, the political and social world “produces” facts: a politician says something, a new policy is introduced, or scientists make an important finding. But there is a twist: malevolent actors make up “alternative facts,” that is, things that have not been said, policies not introduced, and scientific discoveries never made. Both true and false information are reported as (falsifiable) “facts.” Note that an item of “news” in this definition does not have to be a classic report published on a website, but can also be, for example, a social media post, as long as it claims to report something novel to the public.

We may call the entirety of falsifiable information about politically relevant issues, whether true or false, the *population of political news*. Since conservatives and liberals can at least theoretically encounter any politically relevant news, it also seems justified to not restrict this population to a certain subset. Hence, I define the estimands of truth discernment and bias in relation to this population of information. To ask about truth discernment thus means: If someone was exposed to the entire population of political news, true and false, how well would she be able to tell which is which (RQ1)? If someone was exposed to the entire population of political news, how much more would he give credit to ideologically congruent information than to incongruent information (RQ2)? In contrast to studies (implicitly) targeting a balanced population of information, my target population thus takes the real-world supply as a baseline.

One weakness of my conceptualization is that it does not take into account individual *demand*, that is, what kind of information people actually seek out. A longstanding literature on selective exposure tells us that people choose news channels congenial to their worldview (Sears and Freedman 1967; Stroud 2008)—even before the question of whether to believe the information comes up. Thus, an alternative study target could be the “population of self-selected political information,” which would have to be operationalized depending on people’s news diets. Though the present study was not designed to this end, I simulate selective exposure in the results section.

## Data and Methods

### Sampling of News Items

A key component of this study is the selection of news items through sampling from the target population of political news. In an ideal world, we would have a list of all sources publishing such information, and draw a random sample of news items. Of course, this list does not exist. Instead, I rely on the archive of Google News as well as the archives of several fact-checkers as approximations of the universe of political news, both true and false.

Google News is a prominent service that collects news reports from a wide range of sources—at the time of the study, from over 50,000 news organizations globally.^3^ For nine months from October 1, 2019, until May 31, 2020, I collected the entirety of news from the Google News API endpoint https://newsapi.org/v2/everything. I restricted the search to items from US sources that were classified as “general,” “business,” “health,” and “science,” in order to capture politically relevant content. The ensuing collection contained 775,282 items.

Given Google’s pre-selection of authoritative sources, it was unlikely to contain much false news. Thus, my second starting point for data collection were the websites of fact checkers, who have established themselves as organizations devoted to monitor the universe of misinformation (Spivak 2011; Graves 2016). Their archives provide access to true and false news that were fact-checked. To avoid a distorted selection of checked news (Marietta 2019), I excluded fact checkers that only publish false results. I further excluded fact checkers that primarily check speeches by politicians. This left me with the archives of “Snopes,” “PolitiFact,” and “Truth or Fiction.” From their websites, I scraped fact checks along with the original news items for the same period. I only included items that were clearly labeled as either completely true or completely false, and ended up with a set of 710 items (see Supplementary Material section B.2 for details). Note that starting from these two collections does *not* balance the selection of items in terms of truth, as both may contain true and false items. An obvious weakness of relying on fact checkers is that their archives are not very comprehensive, as we can see by the small number of items compared to the Google News collection.

As detailed in the pre-analysis plan,^4^ I simulated that a sample of 40 items from each collection would be sufficient to make it representative of a larger population of items. Evidently, the two collections taken together only approximate the target population in that they are not exhaustive; and in that they contain items *outside* the target population, such as opinion pieces (which are not falsifiable) or non-political articles. Hence, a simple random sample to obtain 40 items out of each collection was not appropriate. Instead, I used an iterative procedure: for both collections, I first randomly sampled 40 items without replacement and excluded items that were not part of the target population or came with other practical problems (for the detailed list of exclusion criteria, see Supplementary Material section B.3). In the second iteration, I then sampled the number of items excluded in the first round, and again excluded items not meeting the requirements; and so on, until I had two samples of 40 items each.

Of the fact-checked items, 9 were labeled as true, 31 as false by the fact-checkers. I fact-checked the items from Google News myself and found that all were true. Thus, of the 80 items, 31 were false, and 49 were true. I next pre-measured the ideological valence^5^ of the news items with raters from MTurk and Prolific (raters were balanced in terms of ideology). These pre-tests were run between June 19 and 30, 2020. As surveys on these platforms are based on opt-in, no response rates can be calculated. Each participant received a random subset, in random order, of 10 items out of the complete set of 80. In total, 200 raters completed the pre-test on MTurk, 185 raters on Prolific. For each item they received, participants answered the two questions: “Assume the above information is entirely accurate. Is it—or was it at the time of publishing—more favorable to liberals or conservatives or neither?” and “Assume the above information is entirely accurate. Is it—or was it at the time of publishing—more consistent with the beliefs of liberals or of conservatives or neither?,” both with response scales from “-2 (liberals)” to “2 (conservatives).” Combining participants from MTurk and Prolific, I computed two averages across raters. As can be seen in Supplementary Material section B.5, item-level averages between the two measures, as well as across the two platforms, are highly correlated (Pearson’s r between 0.80 and 0.96). Ideological valence is coded so that a higher value corresponds to more conservative.

**Figure 2.**
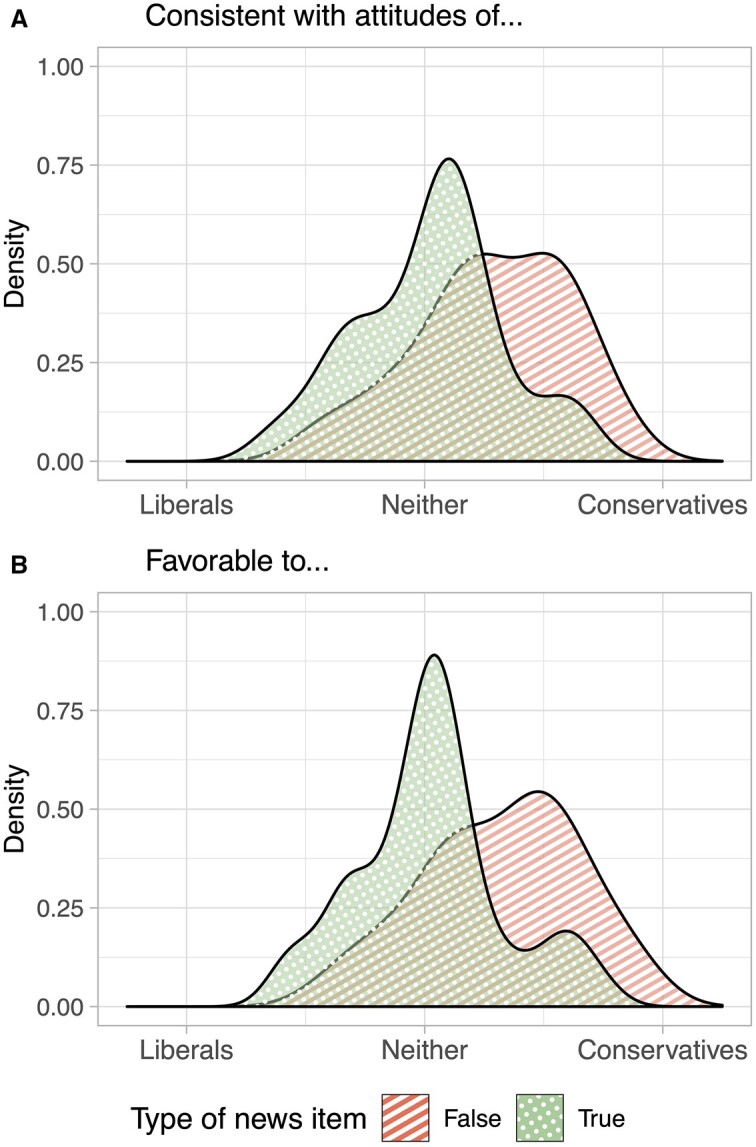
Valence distribution of news items as measured in a pre-test, grouped by item truth. Panel (A) shows density plots of item-level rating averages for whether a news item is “more consistent with the beliefs of liberals or of conservatives or neither.” Panel (B) shows the density for rating average for whether an item is “more favorable to liberals or conservatives or neither.”

Figure 2 shows the distribution of ideological valence of items. Panel (A) shows density plots of item-level average responses to the question of whether a news item is more consistent with the attitudes of liberals or conservatives. Panel (B) shows average responses to the question of whether the information is more favorable to liberals or conservatives. The plots illustrate that false items have a slightly wider range of ideological valence. Further, false items are more often congruent for conservatives than liberals. For the analyses, I will use the “consistence” variable to operationalize valence; robustness checks using the “favorability” item allow identical conclusions (see Supplementary Material section I.2).

### Main Survey

Subsequently, a sample of US respondents recruited through the survey company Dynata took an online survey between June 19 and 30, 2020. Dynata maintains its own actively managed online panel and uses weighted randomization to assign surveys to participants. This means that when panelists enter the platform, a list of potential survey matches is created based on the quota demands and participation limits of surveys. Panelists are then offered a random choice from the list of potential matches. Dynata did not provide cooperation rates for this survey. By enforcing quotas, I ensured that the sample was roughly representative of the US population in terms of gender, age, education, and partisanship, as can be seen in Supplementary Material section C. To further boost external validity, my models use weights based on age, gender, and education computed with R’s anesrake package. After excluding those not compensated by Dynata (i.e., speeders and dropouts, as identified by Dynata), the final sample contained 1,393 subjects. Results hold when including dropouts (Supplementary Material section I.3). The sample size was sufficient to find moderate effect sizes, as determined by power simulations in the pre-analysis plan.

After consenting to participation, respondents were screened in or out according to the quota variables. Next, I asked about their ideology (“Here is a seven-point scale on which the political views that people might hold are arranged from extremely liberal to extremely conservative. Where would you place yourself on this scale, or haven’t you thought much about this?”). Further covariates included partisanship, Facebook use, general media trust, a digital literacy battery (Cronbach’s α: 0.84), an economic policy attitude battery (α: 0.81), a social policy attitude battery (items analyzed separately because of low α;), a need-for-closure battery (items analyzed separately because of low α), a cognitive reflection test score, their ethnicity, state of residence, and income (details in Supplementary Material section D).

Subjects were introduced to the main task with a short explanation and then read 8 news items that were randomly sampled from the 80 news items described above. Subjects saw each item’s original text and headline.^6^ After each item, whether subjects believed the item was measured with the question: “To the best of your knowledge, how accurate is the information in the above item? 0 means not at all, 6 means completely” (Pennycook and Rand 2020).

Since subjects were told at the beginning of the survey that the reports could be true or false, no deception was happening. Nevertheless, to minimize the impact of misinformation exposure, in a debriefing subjects were told which of the items included in the survey were false. The study was approved by the IRB of the European University Institute and pre-registered at http://egap.org/registration/6720. See Supplementary Material section E for a few minor deviations from the pre-registration.

## Results

Are conservatives or liberals better at discerning true from false information (RQ1)? To examine this question, I regress belief scores of encounters between participants and news items (n = 11,125) on an interaction of truth and subject ideology, clustering standard errors at both the subject and the item level, as well as applying weights, in R’s lfe package. The first column of table 1 shows that the interaction is significant. The negative sign implies that the more conservative, the less truth impacts belief. Note that this result holds when using partisanship instead of ideology as the underlying predisposition (cf. Supplementary Material section I.4).

**Table 1.** Linear OLS regressions testing asymmetries in truth discernment and bias.

	Belief					
	RQ1		RQ2		RQ3	
Ideology (-3 to 3)	0.04	(SE = 0.03, *p* = .228)	0.04	(SE = 0.04, *p* = .269)	0.08	(SE = 0.05, *p* = .144)
News item truth (ref: 0)	0.86	(SE = 0.10, *p* = .000)			0.87	(SE = 0.12, *p* = .000)
Ideology * truth	*−*0.09	(SE = 0.10, *p* = .001)			*−*0.11	(SE = 0.06, *p* = .053)
Congruence (ref: 0)			0.14	(SE = 0.06, *p* = .027)	0.23	(SE = 0.12, *p* = .052)
Ideology * congruence			*−*0.14	(SE = 0.06, *p* = .019)	*−*0.13	(SE = 0.09, *p* = .151)
Congruence * truth					*−*0.16	(SE = 0.13, *p* = .226)
Ideology * congruence * truth					0.10	(SE = 0.11, *p* = .361)
Constant	2.59	(SE = 0.09, *p* = .000)	3.16	(SE = 0.08, *p* = .000)	2.61	(SE = 0.11, *p* = .000)
Observations	10,431		7,234		7,234	
R2	0.05		0.01		0.06	

*Note:* Dependent variable across models is the belief score of a subject-news item encounter. All models use clustered standard errors at both the subject and the item level and apply weights (age, gender, education). *P*-values refer to two-tailed tests. In model RQ1, the negative interaction of truth and subject ideology suggests that truth matters less for more conservative participants. In model RQ2, the negative interaction of congruence and subject ideology suggests that congruence matters more for liberals. Model RQ3 does not find any support for a three-way interaction.

**Figure 3.**
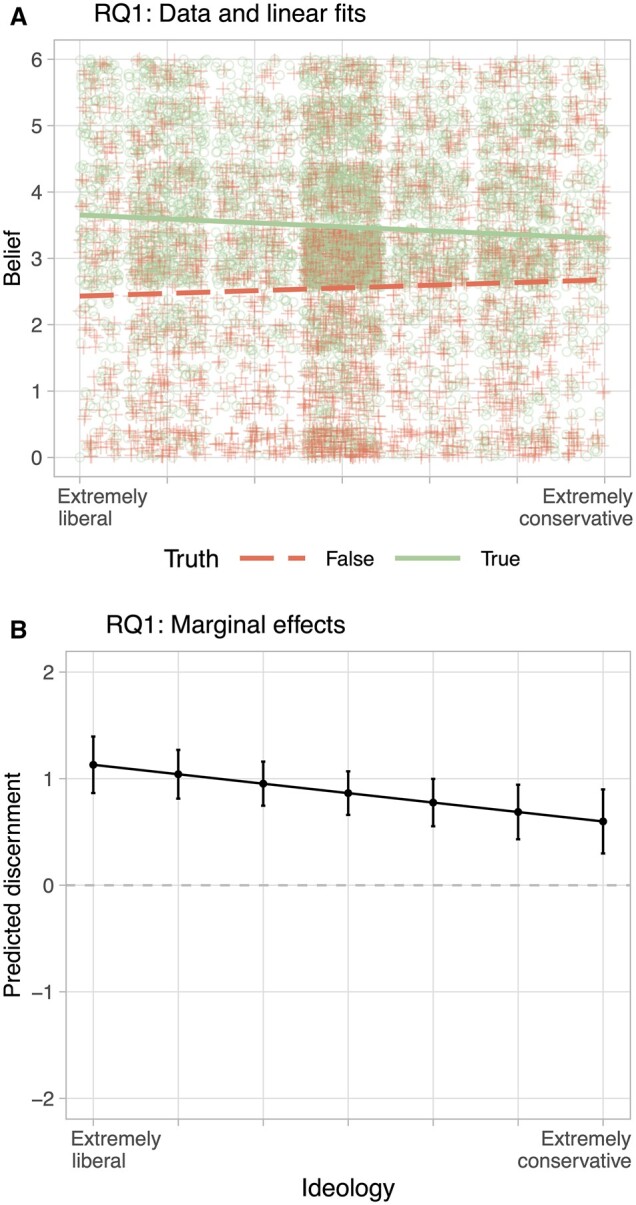
Asymmetries in truth discernment. Panel (A) plots belief of news item against subject ideology, grouped by truth of item. The linear fits illustrate that truth discernment is smaller for conservatives. Panel (B) plots predicted discernment based on the interaction of individual ideology and item truth, as estimated in model RQ1 in table 1.

Figure 3 illustrates this asymmetry between liberals and conservatives. Panel (A) plots the raw data as well as linear fits of belief on ideology, separately for true and false items. Generally, subjects *are* able to tell true from false news, even though the average discernment is moderate. We also see that discernment gets smaller the more conservative subjects are. The asymmetry is confirmed by panel (B), which plots the marginal effect of truth on belief, as estimated by the regression. Those identifying as “extremely liberal” make a predicted difference of 1.13 between true and false, while “extremely conservative” subjects only make a difference of 0.6.

Although my main interest is in differences between conservatives and liberals on a descriptive level, it is also interesting to explore underlying covariates that may explain this asymmetry. If, for example, truth discernment was driven by cognitive reflection, it would be interesting to see whether the asymmetry between conservatives and liberals in discernment can be entirely explained by differences on this trait. To restrict the range of covariates to explore, I first test on which ones conservatives and liberals differ. As detailed in Supplementary Material section F.1, conservatism is significantly correlated with media trust (r = −0.32), digital literacy (r = −0.11), one need-for-closure item (r = 0.07), age (r = 0.2), and education (r = −0.06). Contrary to some previous studies (e.g., Deppe et al. 2015), cognitive reflection is not correlated to ideology. To understand whether the ideological asymmetry in truth discernment can be attributed to any of these characteristics, I add an interaction of truth with each variable to the main model. Supplementary Material section F.2 shows that none of these terms changes the relationship between ideology and truth discernment fundamentally, although some of the variables have interactions with truth discernment in and of themselves.

As discussed previously, my design allows me to balance the news selection *ex post*. As shown in figure 2, false items in my sample have a valence score that is 0.52 units higher, that is, more conservative, than true items. What if, in the spirit of Pennycook and Rand (2019) balancing the selection of items, we made sure that there was an equal number of false news items congruent to liberals as congruent to conservatives (and the same for true news items)? In Supplementary Material section H, I draw random subsets of the data that are balanced to different degrees. The asymmetry tends to disappear with a greater balance on ideological valence, which is interesting given that other studies based on a balanced sample find conservatives to be less truth-discerning.

RQ2 asked whether conservatives or liberals show greater bias, that is, to what extent the congruence of a news item affects their belief that it is true. To test this, I first construct a categorical congruence variable from ideology of the subject and valence of the news item. It takes a value of one when subject and item ideology are in the same direction, and a value of zero in the opposite case. Again, I run a regression of belief scores in subject-news item encounters on an interaction of congruence and ideology (standard errors clustered at the subject and item level and applying weights). The second column of table 1 shows a significant interaction with a negative sign, which suggests that conservatives are less influenced by congruence when judging the truth. This result holds with a continuous congruence variable (Supplementary Material section I.1) and using partisanship instead of ideology (Supplementary Material section I.4).

**Figure 4.**
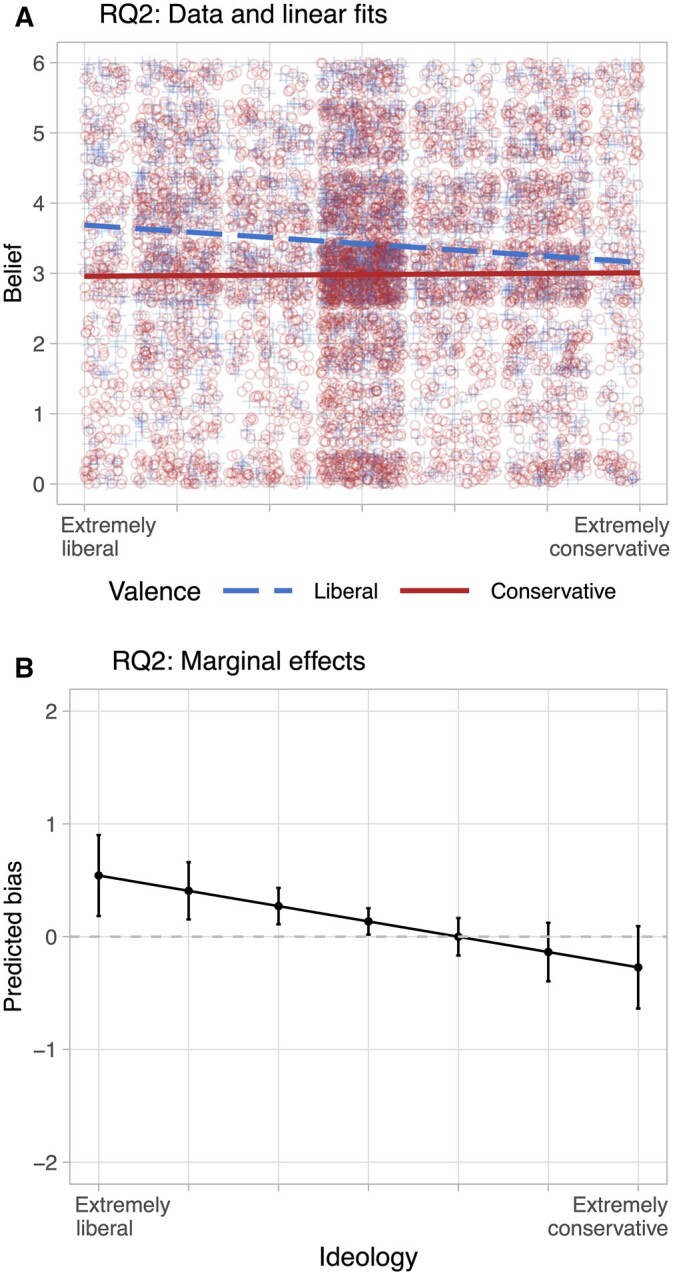
Asymmetries in bias. Panel (A) plots belief of news item against subject ideology, grouped by ideological valence of item. The linear fits illustrate that bias is smaller for conservatives. Panel (B) plots predicted bias based on the interaction of individual ideology and item congruence, as estimated in model RQ2 in table 1.

For graphic illustration, I first show the data in the “raw” form of ideological valence rather than recoded congruence: Panel (A) of figure 4 plots all data points as well as linear fits of the relation between belief and ideology, by valence of news item. Here, valence is a categorical variable so that a news item either has conservative valence if above the scale midpoint, or liberal valence if below the scale midpoint. The plot supports the result of the model: liberals make a greater difference between conservative and liberal information. Panel (B) is another way to look at the result, plotting the marginal effects derived from the regression. The positive effect of congruence—the more congruent a news item, the more likely to be believed—is greatest for liberals, and gets smaller the more to the right we look. The predicted difference for the most conservative subjects suggests that they are not influenced by congruence in their judgments. Note that although it seems that conservatives are slightly more accepting of incongruent than congruent news, this effect is statistically indistinguishable from zero. Again, I examine which covariates could explain these differences, but do not find the asymmetry to change (Supplementary Material section F.2). Finally, I explore how balancing on ideological valence akin to Pennycook and Rand (2019) changes the estimates, finding again that with a balanced sample, the asymmetry in bias tends to disappear (Supplementary Material section H.2).

Finally, RQ3 asked about an interaction between bias and truth discernment: it could be that one ideological group is more truth-discerning, but only if news items are congruent. Or one group could be less biased, but only for false (or true) items. The regression in the third column of table 1 tests a three-way interaction of truth, congruence, and ideology. The coefficient is not significant. Supplementary Material section G visualizes the three-way interaction and suggests that liberal bias is greater for false than true news. As this study was under-powered for detecting small three-way interactions, this intriguing possibility should be explored in the future.

### Exploration: Truth Discernment under Selective Exposure

As briefly discussed, it is well known that citizens are not randomly exposed to political news, but tend to self-select into channels with like-minded content (Stroud 2008). Although my study was not designed to target such a “population of self-selected political news,” it allows me to *simulate* selective exposure by randomly choosing subsamples of the data so that individual news exposures are more ideologically aligned. Specifically, I subsample the data, that is, the 11,125 subject-item encounters, so that all encounters of conservatives with the 40 conservative-leaning items (defined by a median cutoff) are kept, plus their encounters with a random set of 10 liberal-leaning items, and so that all encounters of liberals with all liberal items as well as a random set of 10 conservative items are kept.^7^ I draw 200 such random samples. Each time, I re-estimate truth discernment asymmetries (RQ1). Although these simulations may reconstruct the ideological drivers of selective exposure, they necessarily neglect other individual factors, and also do not measure the *act* of selective exposure. Thus, results need to be interpreted with caution.

**Figure 5.**
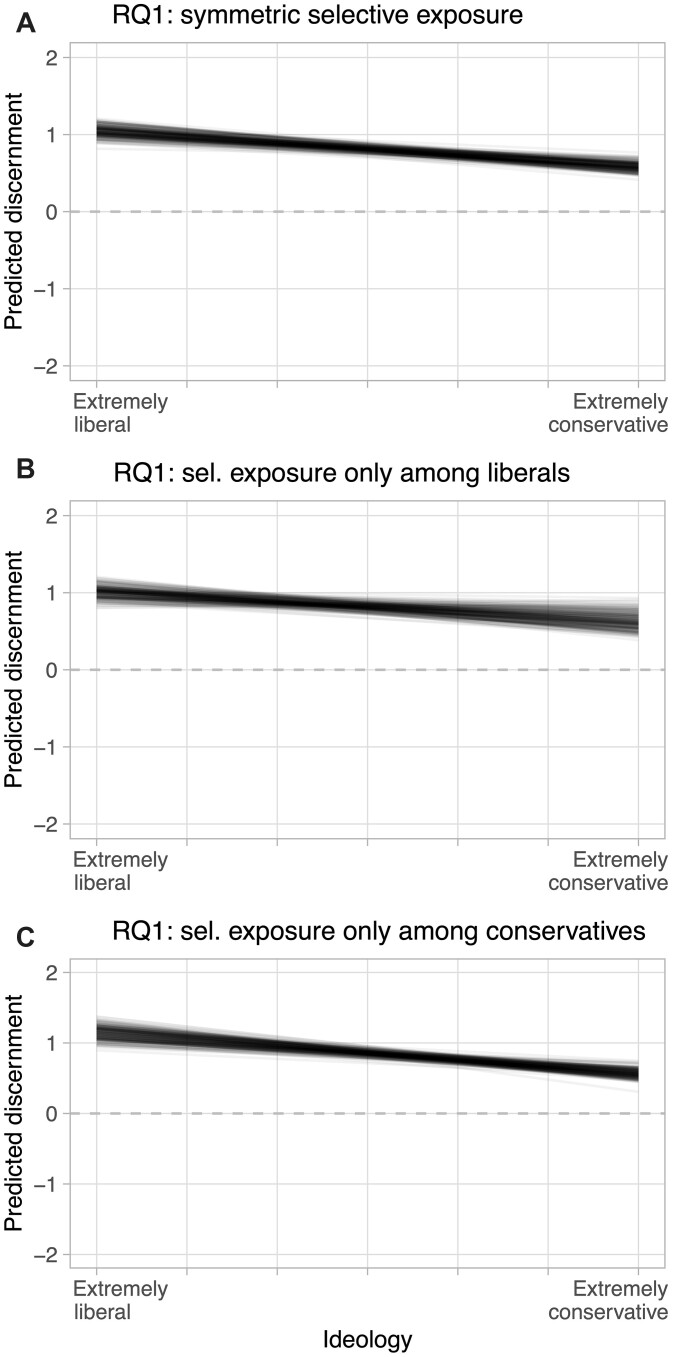
Truth discernment under selective exposure. All three panels show predicted truth discernment by ideology in 200 simulations. In each simulation, news items are re-sampled so that encounters between subjects and items become more aligned ideologically. Panel (A) simulates selective exposure for all subjects. Panel (B) simulates selective exposure only among liberals, Panel (C) only among conservatives.

Figure 5(A) shows marginal effect plots—similar to figure 3(B)—across all 200 simulations. The most important insight: the ideological asymmetry—liberals are more truth-discerning than conservatives—persists under simulated selective exposure. However, it becomes less pronounced: the estimated truth discernment for the most liberal subject, averaged over all simulations, is lower (1.05) than previously (1.13), while it does not change much for the most conservative (0.58 versus previously 0.6).

Why is that? Reconsider the ideological valence of false and true news. Because most misinformation in my sample is conservative-leaning, the “mix” of false news, that is, the proportion of false news that is congruent, does not change much for conservatives. In contrast, the share of congruent false news out of false news that liberals see under simulated selective exposure grows a lot more. In other words, liberals now have much more temptation to believe falsehoods. This is an interesting—and somewhat paradoxical—consequence of selective exposure. However, these are simulated results only; future designs should account for selective exposure in a more targeted way.

What is more, the finding that selective exposure decreases liberal discernment has to be qualified in light of shifting *quantities* of misinformation: under simulated selective exposure, liberals on average see 13.75 false items, whereas conservatives see 25. So even though liberals’ truth discernment score may have been reduced, they will *in total* acquire fewer misbeliefs. Once again, this shows how important it is to take the real-world supply into account.

Things get more complicated when we consider that the degree of selective exposure could differ across the ideological spectrum—another prominent question in the literature on asymmetries (Iyengar et al. 2008; Frimer, Skitka, and Motyl 2017). Two additional simulation studies depicted in figure 5 show how discernment changes when only liberals (B), or only conservatives (C), engage in selective exposure. By the same logic explained above, selective exposure has a bigger effect on truth discernment of liberals. Here, too, the caveat is that these results are simulated, and more sophisticated designs would reflect *individual* news diets, rather than assuming identical exposure for all members of a group.

## Conclusion

An increasing body of research suggests that problematic patterns of information processing exist, in nuanced ways, across the ideological space (Eichmeier and Stenhouse 2019; Ecker, Sze, and Andreotta 2021; Guay and Johnston 2022). This study addressed two such patterns for which evidence on asymmetries is inconclusive. First, the (in)ability to discern true from false news, and second, the tendency to give more credit to ideologically convenient information. I found that liberals are more truth-discerning than conservatives, but they also are more prone to bias. However, while these asymmetries exist, they are not drastic in magnitude.

Studying such phenomena presupposes, tacitly, a definition of what kind of information universe one is talking about. Lacking such a definition, it is difficult to see what the selection of news used in a study represents. In addition, an arbitrary selection may yield estimates different from hypothetical alternative selections. This variation of estimates is acceptable when the selection is made in a principled way—ideally, by sampling from a predefined target population. To adhere to these ideas, I built a collection of over 775,000 news items, from which I randomly chose 80 items to test participants’ accuracy judgments.

My findings have to be interpreted in light of this methodology. Most previous studies use a sample of information that is balanced on ideological valence. My study, in contrast, exposes subjects to an item sample meant to mirror the real-world supply. Prior work suggests that misinformation is more likely to cater to conservatives (Guess, Nyhan, and Reifler 2018; Munger 2020). In my sample, too, at least for the time frame in question, misinformation catered more to conservatives, and this contributes to them being on average more likely to believe false information. However, when I balance the information sample *ex post*, similar to Pennycook and Rand (2019), conservatives’ truth discernment gets much closer to that of liberals. This underlines the need for researchers to think clearly about what kind of asymmetries they are estimating, given the stimuli they select.

The finding that liberals are more biased contributes to the debate over whether “bias is bipartisan” (Ditto et al. 2019). The asymmetry hypothesis locates bias on the conservative side (Jost 2017); the symmetry hypothesis expects it across the ideological spectrum (Kahan 2016; Guay and Johnston 2022). In contrast to both, I find evidence for a reverse asymmetry. As the variables often theorized to be responsible for these ideological asymmetries—need for cognition and cognitive reflection—do not correlate strongly with ideology in my sample, I unfortunately cannot shed light on the deeper mechanisms of this asymmetry. I also do not find evidence for the possibility that higher education may explain both liberalism and bias.

My approach to select informational stimuli, although meant to be principled, obviously comes with a range of weaknesses. First, much misinformation flies under the radar, as attested to by the small collection of items resulting from fact-checker archives. This is especially problematic as the selection made by fact checkers themselves is somewhat of a black box (Uscinski and Butler 2013; Nieminen and Rapeli 2019). All three fact-checking organizations stress their bipartisan approach, but some bias in determining “checkworthiness” is likely unavoidable.

As it is not easy to get at the universe of misinformation, future work will have to explore original ways to do so. One recent study exploited RSS feeds from lists of untrustworthy websites to obtain a comprehensive picture of all false news (Godel et al. 2021). Similarly, rather than relying on collecting individual misinformation items like I did, one could sample articles from a list of untrustworthy sites (Allcott, Gentzkow, and Yu 2019). However, this would require the additional step of ascertaining whether items are true or false.

Although my study has its strengths in terms of “stimulus validity,” it has drawbacks in terms of “temporal validity” (Munger 2019). This refers to the idea that as we draw inferences from historical data, we cannot easily apply them to the present or future. Selecting many stimuli like I did, despite mirroring the real-world supply, is arguably more affected by this issue than selecting fewer, but more carefully curated items, that is, the approach taken by Pennycook and Rand (2019). My approach may be a better snapshot of the information environment at the time, but it may not travel temporally as well as a design that balances stimuli on a number of dimensions known to matter today and tomorrow. Ultimately, this project illustrates that there is a spectrum of alternative selection strategies when researching news processing—and hopefully, will inspire researchers in the future to approach this spectrum in innovative ways.

## Supplementary Material

nfad013_Supplementary_Data

## Data Availability

Replication data and documentation are available at https://osf.io/82w7u/.

## Footnotes

1 The literature knows different terms for this phenomenon, such as “confirmation bias” or “assimilation bias.” For brevity, I stick to “bias.”

2 Note that this variability of results also emerges for a re-sampling specification that ensures that not only the number of pro-Democrat and pro-Republican items are the same, but also their respective distance to the valence midpoint (see Supplementary Material section A).

3
https://newsapi.org/, accessed 5 May 2020.

4
https://osf.io/82w7u/.

5 I refer to “valence” as the variable independent of someone’s individual ideology. “Congruence” is the variable that indicates to what degree ideological valence and someone’s ideology match.

6 The study contained two experimental conditions that are not analyzed here. One of them pertained to the presentation of items: for half the subjects, items were accompanied by a screenshot of the original article and emphasized the source. This treatment did not affect the outcomes tested.

7 The average number of encounters in the 200 re-samples is 6,580. See replication code for details.
