# Supplementary material for: Truth and Bias, Left and Right: Testing Ideological Asymmetries with a Realistic News Supply
Source: Public Opin Q. 2023 Apr 29;87(2):267–92. doi: 10.1093/poq/nfad013 (PMC10371040; doi:10.1093/poq/nfad013)
Supplement: nfad013_Supplementary_Data [file nfad013_supplementary_data.pdf]

Truth and Bias, Left and Right. Testing ideological asymmetries with a realistic news supply

SUPPLEMENTARY MATERIALS

Bernhard Clemm von Hohenberg*

15 February, 2023

Contents

A Pennycook & Rand (2019) replication **2**

B News item sample **3**

B.1 Data collection from Google News 3

B.2 Data collection from fact-checkers 3

B.3 Iterative sampling exclusions 4

B.4 Final item samples 10

B.5 Ideological valence pre-testing 15

C Subject sample **24**

D Variable measurement **27**

E Differences to pre-registration **30**

F Ideological asymmetries and covariates **31**

F.1 Correlations of ideology and covariates 31

F.2 Regressions with covariates 32

G Plot of truth discernment-bias interaction (RQ3) **36**

H Re-estimating truth discernment with a balanced item sample **37**

H.1 Truth discernment 37

H.2 Bias 38

*ASCoR, University of Amsterdam. Email b.f.d.clemm@uva.nl. Orcid ID 0000-0002-6976-9745. This Supplementary Materials file is based on a fully reproducible RMarkdown file that contains code to replicate all graphs and analyses, which can be found at `https://osf.io/82w7u/`.

I Robustness checks **39**
I.1 Continuous congruence variable . . . 39
I.2 Favorability instead of consistence . . . 40
I.3 Including subjects who did not finish . . . 41
I.4 Partisanship instead of ideology . . . 41

A Pennycook & Rand (2019) replication

The authors of the original study did not only ensure that there was an equal number of pro-Clinton and pro-Trump items. They also balanced on the continuum of ideological valence, so that pro-Clinton items were pretested as equally distant from the ideology midpoint as pro-Trump items (as communicated in an email exchange). To account for this, I filter out sub-samples that do not meet this criterion in the below plot. Of my 500 sub-samples, 0.94 are balanced this way. Of those, only 0.53 are significant, supporting the point made in main paper.

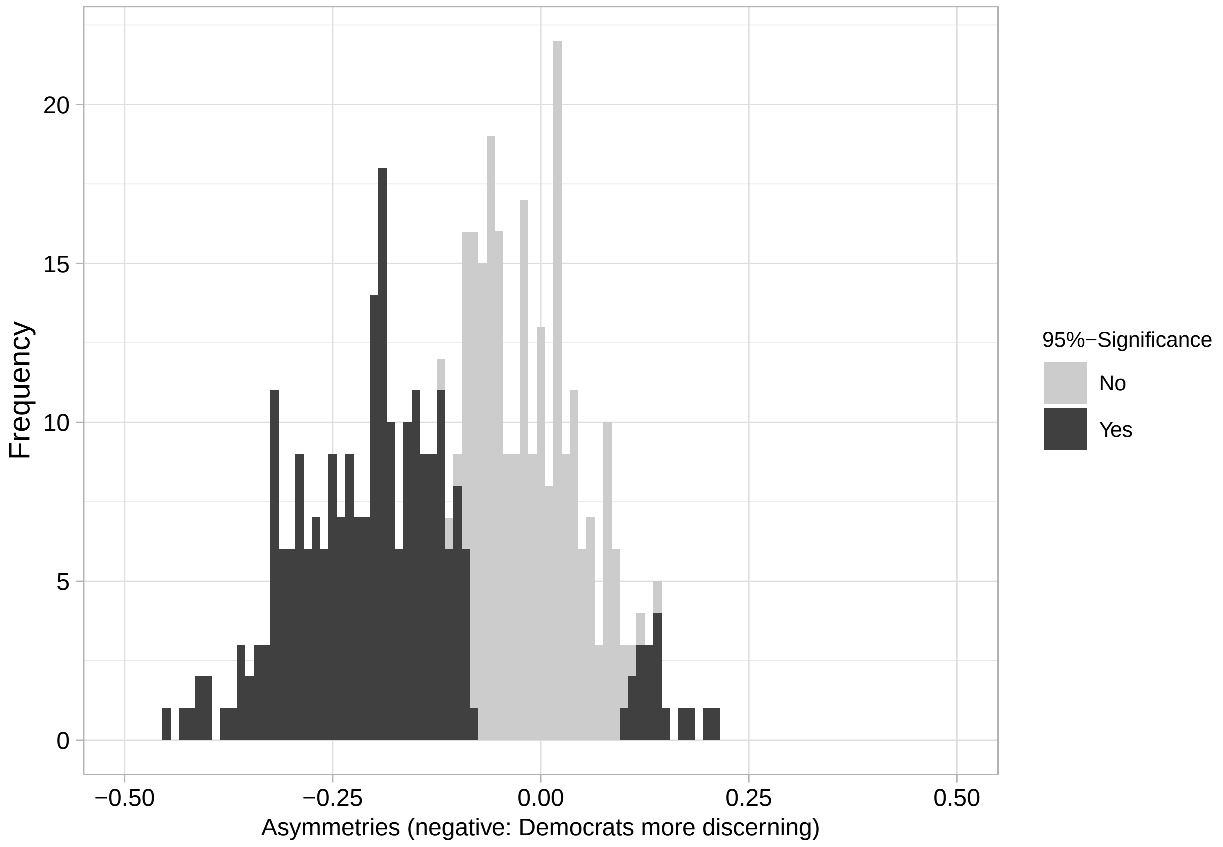

Figure 1: Re-analyis of Pennycook and Rand (2019), with continuous balancing

B News item sample

B.1 Data collection from Google News

The Google News data was collected through the Google News API with the following parameters: "from" and "to" in order to define the time frame; "language" set to "en" to limit results to English news items; "sources" to a list of sources determined through the endpoint `https://newsapi.org/v2/sources` with parameter "country" set to "us" and "category" to "business", "general", "health" or "science" to exclude news that are likely non-political.

B.2 Data collection from fact-checkers

A first risk in relying on fact-checkers is a potential ideological bias in selection what information to check (Ostermeier 2011; Uscinski and Butler 2013; Marietta, Barker, and Bowser 2015). Second, some fact-checker only publish about news checked as false. The aim of publishing only the latter might distort the selection process (Marietta 2019). A third risk is that although fact-checkers have been found to be reliable on outright truths and falsehoods, ratings of ambiguous claims are more unreliable (Nieminen and Rapeli 2019; Lim 2018). To minimize these risks, I excluded fact-checkers that only publish false results. I further excluded fact-checkers that primarily check speeches by politicians. I ended up with the three organizations Snopes, Politifact and Truth or Fiction.

The risk of selection bias cannot be excluded completely. At least in theory, all three organisations commit to non-partisan selection, although procedures do not seem formalized. Snopes explains that they "don't choose or exclude items for coverage based on whether they deal with Republican/Democratic, conservative/liberal, or religious/secular issues" and that "reader interest" plays a role.[1] PolitiFact lists several selection criteria, among them whether a statement is "significant" and commits "to check from both Democrats and Republicans".[2] Truth or Fiction describes their focus as "on stories that are the most widely-circulated via social media".[3]

The fact-check collection was composed through scraping the archives of Snopes, Politifact and Truth or Fiction for the same time frame with the following parameters:

- `https://www.snopes.com/fact-check/category/politics/`; reports labelled as "mixture", "unproven", "outdated", "miscaptioned", "scam", "legend", "labeled satire", "lost legend", "mostly true" and "mostly false" were not included; those labelled as "false", "misattribution", "true" or "correct attribution" were included.
- `https://www.politifact.com/factchecks/`; categories "Facebook Post", "Bloggers" and "Instagram" included; "Viral Image" and "Names" exluded; reports labelled as "True", "False" or "Pants on Fire" included, "Mostly True", "Half True" and "Mostly False" excluded.

[1] https://www.snopes.com/faq/decide-fact-check/, accessed August 24th, 2020.

[2] https://www.PolitiFact.com/article/2018/feb/12/principles-truth-o-meter-PolitiFacts-methodology-i/, accessed August 24th, 2020.

[3] https://www.truthorfiction.com/about/, accessed August 24th, 2020.

- `https://www.truthorfiction.com/category/fact-checks/politics/`; items labelled “Truth!” or “Fiction!” included,“Reported to be Truth!”, “Reported to be Fiction!”, “Unproven!” and “Truth! & Fiction!” excluded.

Below, Section B.3 list the items excluded during the iterative sampling procedure, separately for the two collections. Exclusion reasons as explained in the paper. Section B.4 lists the URLs of the items in the final sample. Each item has a unique ID that corresponds to the ID in Table 5.

B.3 Iterative sampling exclusions

I filtered out items according to the following criteria, as listed in the pre-analysis plan. To meet the definition of the population of political news, I excluded items (1) that have a normative character, such as analysis or opinion pieces; (2) items that were non-political in content; and (3) information that consisted only of a social media statement by a politician that was not embedded in a report. Further, the practical limitation of a survey required to exclude (4) items that consist of summaries of several news reports, such as press digests or “latest updates”, as they would complicate the measurement of the main outcome; (5) overly long items such as long-read features, if it was impossible to shorten them without distorting the substance (otherwise, items were shortened if too long for the survey format); (6) items that consisted primarily of video, photo or audio content, also including graphic memes; (7) items that were primarily reactions to other reports that could not be meaningfully represented without including the reference; and (8) items with broken URLs or behind paywalls.

Table 1: Sampling exclusions Google News collection

Iteration	Item URL	Reason for exclusion
1	www.cbsnews.com/video/remains-of-pearl-harbor-sailor-return-home/	Video/audio/photo
1	www.foxnews.com/opinion/tara-mcclary-reeves-thank-service-veterans	Opinion/analysis
1	www.foxnews.com/entertainment/dennis-quaid-laura-savoie-engagement-ring	Non-political
1	feedproxy.google.com/~r/discovercrux/~3/ua-_f7ICGVg/when-a-bee-falls-in-water-it-surfs-tiny-ripples-to-safety	Broken URL
1	www.aljazeera.com/indepth/features/diplomats-sale-ambassadorship-bought-lost-191202080408558.html	Length
1	www.washingtonpost.com/world/asia_pacific/pakistani-lawyer-who-fought-for-unlawfully-held-is-abducted/2019/12/17/4871a99e-20b5-11ea-b034-de7dc2b5199b_story.html	Length
1	www.msnbc.com/kasie-dc/watch/the-nixonian-smoking-gun-from-trump-impeachment-probe-71203909720”	Video/audio/photo
1	www.politico.com/news/2019/12/10/democrats-fret-trade-deal-trump-reelection-081447	Opinion/analysis
1	www.usatoday.com/story/news/nation/2020/03/22/coronavirus-n-95-mask-shortage-us-fema-donald-trump/2895344001/	Length
1	apnews.com/f3de10fe7d622b0a5b06e0969975fa2b	Non-political
1	disneyparks.disney.go.com/blog/2019/10/disneyland-resort-cast-members-help-build-12th-disney-sponsored-kaboom-playground-in-anaheim/	Non-political
1	www.businessinsider.com/ces-2020-bosch-virtual-visor-blocks-sun-glare-while-driving-2020-1	Non-political
1	abcnews.go.com/Health/video/coronavirus-daily-update-06-2020-70543470	Video/audio/photo
1	radio.foxnews.com/2020/03/19/a-look-inside-facebook-with-steven-levy/	Video/audio/photo
1	www.washingtonpost.com/opinions/2019/10/24/what-jews-think-about-anti-semitism/	Opinion/analysis
1	www.newsweek.com/impeachment-trial-1483970	Opinion/analysis
1	time.com/5803809/arnold-schwarzenegger-coronavirus/	Video/audio/photo
1	www.reuters.com/article/us-rio-tinto-aluminium-iceland-idUSKBN2060Y8	Non-political
1	www.reuters.com/article/usa-stocks-idUSZXN0RP62I	Summary article
1	www.cnn.com/2019/11/30/entertainment/kpop-pressures-goo-hara-sulli-intl-hnk-scli/index.html	Non-political
1	apnews.com/f95ab09060ec2ff4b0aa48f725abd224	Non-political

Iteration	Item URL	Reason for exclusion
1	www.nbcnews.com/politics/trump-impeachment-inquiry/live-blog/impeachment-live-updates-judiciary-debates-articles-impeachment-n1100121	Summary article
1	www.businessinsider.com/kobe-bryants-last-public-appearance-nba-game-with-daughter-gigi-2020-1	Non-political
1	www.wsj.com/articles/u-s-indicts-venezuelan-president-nicolas-maduro-on-allegations-of-drug-trafficking-11585236443	Paywall
1	www.businessinsider.com/jane-street-millennium-corporate-bond-trading-technology-2020-1	Non-political
1	www.newsweek.com/how-some-senators-positions-impeachment-trial-witnesses-changed-clinton-trump-1482192	Length
2	www.reuters.com/article/emerging-markets-idUSL8N29P1IB	Summary article
2	www.foxnews.com/science/astronaut-scott-kelly-describes-life-coronavirus-lockdown	Non-political
2	feedproxy.google.com/~r/apartmenttherapy/thekitchn/~3/wp0NyzrWVdQ/a-week-of-easy-make-ahead-comfort-meals-23017865	Broken URL
2	apnews.com/d665ac3c89877273c67ea9c8cae65b23	Non-political
2	feedproxy.google.com/~r/crunchyroll/animenews/~3/dqyzJoQ0bRs/xenoblade-chronicles-gears-up-for-switch-debut-with-overview-trailer	Broken URL
2	feedproxy.google.com/~r/macupdate/~3/WvMasE4yNqM/1581154616	Broken URL
2	rss.cnn.com/~r/services/podcasting/studentnews/rss/~3/EQEpyrIWIRI/ten-0325.cnn_3157044_ios_1240.mp4	Broken URL
2	thehill.com/business-a-lobbying/lobbying-hires/482673-lobbying-world	Non-political
2	www.nbcnews.com/news/crime-courts/patrick-frazee-s-ex-girlfriend-reveals-kelsey-berreth-s-last-n1078236	Non-political
2	www.foxnews.com/opinion/biden-sex-assault-allegation-media-hypocrisy-kavanaugh-tim-graham	Opinion/analysis
2	mmajunkie.usatoday.com/2019/10/ufc-243-free-fight-relive-fight-of-the-year-candidate-between-israel-adesanya-and-kelvin-gastelum	Non-political
2	www.foxnews.com/sports/ohio-state-ryan-day-calls-fiesta-bowl-clemson	Non-political
3	www.cnn.com/videos/world/2019/10/23/protests-lebanon-chile-hong-kong-orig-md-mss.cnn	Video/audio/photo
3	www.cbsnews.com/news/jose-andres-pitch-baseballl-world-series-washington-nationals-booing/	Non-political

Iteration	Item URL	Reason for exclusion
3	www.usatoday.com/videos/sports/golf/whatimhearing/2020/03/13/coronavirus-affects-premiere-golf-tournament/5047861002/	Non-political
3	www.businessinsider.com/personal-finance/student-loan-forbearance-vs-deferment-coronavirus-2020-5	Non-political
3	in.reuters.com/article/ubs-group-russia-moves-idINKBN22O1OX	Non-political
3	www.businessinsider.com/power-moves-of-the-week-intel-shake-shack-credit-suisse-2019-10	Non-political
3	apnews.com/543fc8d6fe36c0f181ad14a85d704f03	Non-political
4	www.foxnews.com/sports/conor-mcgregor-holds-all-the-ufc-cards-after-comeback-win	Non-political
4	www.cnn.com/2019/11/24/investing/stocks-week-ahead/index.html	Non-political
4	feedproxy.google.com/~r/allcnetvideopodcasts/~3/6F-0Wh6dHPs/NBC_Streaming_v2_SITE_234422_740.mp4	Broken URL
5	apnews.com/3011e7f34c8ef5a5be9a78ae2a992183	Non-political
5	ca.reuters.com/article/businessNews/idCAKBN1WU148-OCABS	Non-political
6	www.cbsnews.com/video/apple-ceo-files-brief-at-supreme-court-supporting-daca/	Video/audio/photo
6	www.bloomberg.com/news/audio/2019-10-18/tesla-plays-gravity-in-woods-high-flying-ark-etf-podcast	Video/audio/photo
7	www.reuters.com/article/us-tennis-wtafinals-idUSKBN1X91S0	Non-political
8	www.reuters.com/article/us-bank-of-america-leadership-idUSKBN20C2EB	Non-political
8	ca.reuters.com/article/technologyNews/idCAKBN1Y217X-OCATC	Non-political

Table 2: Sampling exclusions fact-checks collection

Iteration	Item link	Reason for exclusion
1	www.politifact.com/factchecks/2020/feb/20/blog-posting/death-hoax-about-kobe-bryants-eldest-daughter-spre/	Non-political
1	www.politifact.com/factchecks/2020/jan/06/facebook-posts/soldiers-photo-are-traveling-break-basic-training-/	Video/audio/photo
1	www.politifact.com/factchecks/2019/nov/26/blog-posting/dont-fall-story-mall-santa-beating-child-molester/	Non-political

Iteration	Item link	Reason for exclusion
1	www.politifact.com/factchecks/2020/apr/09/facebook-posts/photos-show-money-streets-venezuela-2019-not-italy/	Video/audio/photo
1	www.politifact.com/factchecks/2019/nov/11/facebook-posts/no-craigslist-ad-offering-pay-trump-support-phony/	Video/audio/photo
1	www.snopes.com/fact-check/trump-discover-know-everything/	Video/audio/photo
1	www.snopes.com/fact-check/white-house-trump_photo-delete/	Video/audio/photo
1	www.snopes.com/fact-check/experts-officials-pandemic-2018/	Reference to other article
1	www.politifact.com/factchecks/2019/dec/10/facebook-posts/celebrity-death-hoax-about-will-smith-and-his-son-/	Non-political
1	www.politifact.com/factchecks/2020/apr/27/facebook-posts/no-proof-mosquitoes-transfer-covid-19-between-peop/	Non-political
1	www.snopes.com/fact-check/buttigieg-dogs-clipping/	Video/audio/photo
1	www.politifact.com/factchecks/2020/feb/29/facebook-posts/no-nancy-pelosi-was-not-escorted-out-restaurant-dr/	Video/audio/photo
1	www.snopes.com/fact-check/joe-biden-corn-dog/	Video/audio/photo
1	www.politifact.com/factchecks/2020/apr/23/facebook-posts/no-democrats-arent-pushing-microchips-fight-corona/	Opinion/analysis
1	www.politifact.com/factchecks/2020/feb/27/blog-posting/no-bernie-sanders-did-not-collaborate-marxist-regi/	Video/audio/photo
1	www.politifact.com/factchecks/2020/feb/20/facebook-posts/starbucks-exec-didnt-say-she-dislikes-white-people/	Reference to other article
1	www.snopes.com/fact-check/trump-biden-coffin/	Video/audio/photo
1	www.politifact.com/factchecks/2019/sep/27/facebook-posts/greta-thunberg-did-not-pose-isis-or-george-soros/	Video/audio/photo
1	www.snopes.com/fact-check/pat-robertson-shooting-up-churches/	Video/audio/photo
2	www.politifact.com/factchecks/2020/mar/23/blog-posting/no-such-thing-free-lowes-coupon-answering-some-que/	Non-political
2	www.snopes.com/fact-check/james-clapper-barack-obama-russia/	Video/audio/photo
2	www.snopes.com/fact-check/monica-lewinsky-trump-tweet/	Video/audio/photo

Iteration	Item link	Reason for exclusion
2	www.politifact.com/factchecks/2019/dec/10/facebook-posts/social-posts-falsely-connect-trumps-impeachment-bi/	Opinion/analysis
2	www.snopes.com/fact-check/trump-covid19-statements/	Reference to other article
2	www.politifact.com/factchecks/2020/apr/28/facebook-posts/uv-radiation-not-common-way-kill-viruses-and-bacte/	Video/audio/photo
2	www.snopes.com/fact-check/trump-ever-voted-by-mail/	Reference to other article
2	www.snopes.com/fact-check/trump-live-let-die-song-mask/	Video/audio/photo
2	www.truthorfiction.com/ivanka-trump-and-the-alexis-de-tocqueville-quote/	Reference to other article
2	www.politifact.com/factchecks/2020/mar/23/facebook-posts/no-your-census-response-wont-affect-whether-you-ge/	Non-political
2	www.truthorfiction.com/did-mike-bloomberg-say-bernie-sanders-would-have-beaten-trump-in-2016/	politician quote
2	www.politifact.com/factchecks/2020/jan/29/blog-posting/no-msnbc-reporter-was-not-fired-over-viral-live-ai/	Broken URL
3	www.politifact.com/factchecks/2019/oct/30/facebook-posts/no-evidence-cream-tartar-orange-juice-drink-will-h/	Non-political
3	www.politifact.com/factchecks/2019/nov/15/facebook-posts/no-sex-traffickers-arent-using-zip-ties-mark-victi/	Video/audio/photo
3	www.politifact.com/factchecks/2020/jan/02/facebook-posts/social-media-post-recycles-debunked-quotes-joe-bid/	politician quote
3	www.politifact.com/factchecks/2020/feb/29/facebook-posts/no-mountain-dew-isnt-being-canceled-being-bad-you/	Non-political
3	www.politifact.com/factchecks/2020/apr/13/tweets/2016-video-trump-urging-sick-people-vote-resurface/	Video/audio/photo
3	www.politifact.com/factchecks/2020/mar/03/facebook-posts/viral-image-draws-misleading-parallel-between-coro/	Video/audio/photo
4	www.snopes.com/fact-check/tulsi-gabbard-impeachment-vote/	

Iteration	Item link	Reason for exclusion
4	www.politifact.com/factchecks/2019/nov/08/facebook-posts/post-obama-being-furious-about-photo-him-holding-p/	Reference to other article
4	www.politifact.com/factchecks/2020/jan/23/facebook-posts/there-outbreak-china-wuhan-coronavirus-there-not-v/	Video/audio/photo
4	www.politifact.com/factchecks/2020/jan/13/facebook-posts/no-viral-post-isnt-correct-australias-gun-laws-vio/	Video/audio/photo
5	www.truthorfiction.com/mods-are-asleep-post-mike-bloomberg-in-epsteins-black-book/	Video/audio/photo
5	www.truthorfiction.com/are-these-really-donald-trumps-notes/	Video/audio/photo
5	www.truthorfiction.com/andrew-yang-pathway-to-citizenship-make-them-earn-it-policy-statement/	Video/audio/photo
6	www.politifact.com/factchecks/2019/oct/01/blog-posting/clint-eastwood-still-not-dead/	Non-political
6	www.truthorfiction.com/what-is-a-scif-and-why-does-it-matter/	Video/audio/photo

B.4 Final item samples

Table 3: Final sample of Google News items

URL	Item ID
https://www.breitbart.com/entertainment/2020/04/08/don-cheadle-another-trump-presidency-could-end-us-all/	1
https://thehill.com/blogs/blog-briefing-room/news/466023-bulgaria-coach-sorry-for-racist-chants-six-people-detained	2
https://www.nbcnews.com/politics/trump-impeachment-inquiry/gop-senators-under-pressure-witness-testimony-trump-trial-after-bolton-n1123776	3
https://www.reuters.com/article/us-pemex-results-idUSKCN20L2OG	4
https://abcnews.go.com/US/wireStory/aclu-appeals-case-alleging-wv-trans-teen-harassment-69289986	5
https://apnews.com/6be38b474bd64dd6ed7a839294c555cc	6
https://www.businessinsider.com/healthcare-workers-coronavirus-challenges-housing-apartments-2020-4	7
https://abcnews.go.com/International/wireStory/uk-economy-shrinks-quarter-full-lockdown-70652099	8

Table 3: Final sample of Google News items *(continued)*

URL	Item ID
https://www.breitbart.com/tech/2020/05/08/u-of-wisconsin-madison-furloughs-employees-despite-accepting-10-million-in-coronavirus-relief/	9
https://www.businessinsider.com/how-nsos-platform-is-helping-countries-track-the-coronavirus-2020-4	10
https://apnews.com/613a79beaf94ab2943bb9cc91ca61788	11
https://apnews.com/1dd4abccb2914fdc95357465dcbca089	12
https://apnews.com/6f004b80dfb84f85934b51c00e92a9ab	13
https://www.nbcnews.com/health/health-news/we-re-not-there-yet-key-steps-reopen-economy-fauci-n1183606	14
https://uk.reuters.com/article/uk-health-coronavirus-swiss-idUKKBN21G0AS	15
https://www.foxnews.com/media/former-acting-ag-calls-upcoming-ig-report-most-consequential-report-in-many-years	16
https://www.foxnews.com/media/white-house-trade-navarro-trump-jobs-report	17
https://www.reuters.com/article/us-china-health-australia-idUSKBN1ZT0K4	18
https://abcnews.go.com/US/explosion-erupts-texas-chemical-refinery/story	19
https://thehill.com/policy/defense/467290-nuclear-command-nominee-sidesteps-questions-on-whether-to-stay-in-arms-control	20
https://uk.reuters.com/article/uk-cambodia-politics-idUKKBN1XL0AQ	21
https://www.nbcnews.com/news/us-news/family-wichita-man-killed-police-swatting-incident-seeking-25-million-n1079836	22
https://www.reuters.com/article/us-usa-trump-whistleblower-australia-idUSKBN1WH001	23
https://www.reuters.com/article/us-health-coronavirus-indonesia-cases-idUSKBN22U12J	24
https://apnews.com/b7c48507edd97d13a7f3ab32aeb0815b	25
https://www.newsweek.com/donald-trump-jr-accuses-democrats-using-gestapo-tactics-calls-impeachment-proceedings-1469733	26
https://apnews.com/4f45cf311abb16d3c9f0b012090e534b	27
https://apnews.com/9ab735616b5c783faf58ec005394b765	28
https://www.washingtonpost.com/politics/2020/04/02/wealthy-cities-have-more-coronavirus-cases-new-york-poorer-communities-are-more-affected/	29
https://www.reuters.com/article/us-health-coronavirus-regeneron-pharms-idUSKCN21R2WN	30

Table 3: Final sample of Google News items *(continued)*

URL	Item ID
https://thehill.com/policy/finance/471533-harris-waters-release-100-billion-affordable-housing-bill	31
https://apnews.com/99ce3a06c7f12dda3e9cdbb083b81eb8	32
https://www.reuters.com/article/us-health-coronavirus-gilead-pricing-idUSKBN22I2NM	33
https://thehill.com/homenews/administration/492106-white-house-reporter-tests-negative-for-coronavirus	34
https://af.reuters.com/article/worldNews/idAFKBN1WW17J	35
https://www.reuters.com/article/us-russia-shooting-casualties-idUSKBN1YN2DK	36
https://in.reuters.com/article/us-health-coronavirus-stimulus-banks-exc-idINKBN21K075	37
https://apnews.com/8725deb9606e4f54939695afb6e61093	38
https://www.reuters.com/article/us-pakistan-pollution-idUSKBN1XW1G4	39
https://apnews.com/6bdd7209ec32f68868b5aac8f08f13bd	40

Table 4: Final sample of fact-checked items

URL	Item ID
https://www.newsweek.com/ice-fails-redact-document-reveals-location-urban-warfare-training-facility-1458732	41
https://www.facebook.com/WarriorCodeNation/photos/a.1725067604416925/2591241537799523/	42
https://www.theguardian.com/politics/2019/dec/11/boris-johnson-hides-in-fridge-to-avoid-piers-morgan-interview	43
https://web.archive.org/web/20190616072052/https://newspunch.com/nicole-kidman-blacklisted-hollywood-support-president/	44
www.truthandaction.org/un-plans-implant-everyone-biometric-id/2/	45
http://archive.is/M7hGS	46
https://www.dailymail.co.uk/news/article-8085175/US-hospitals-prepare-96-MILLION-coronavirus-infections-HALF-MILLION-deaths.html	47
https://www.newsweek.com/video-resurfaces-michelle-obama-praising-wonderful-human-being-harvey-weinstein-after-sex-abuse-1488875	48

Table 4: Final sample of fact-checked items *(continued)*

URL	Item ID
https://breakingnewshouse.com/2019/09/06/actor-will-smith-im-not-afraid-to-say-that-trump-and-his-supporters-are-ba/	49
https://edition.cnn.com/2020/01/16/us/missouri-parental-library-oversight-bill-trnd/index.html	50
Re-constructed from https://www.snopes.com/fact-check/bloomberg-political-ads/	51
https://www.facebook.com/photo.php	52
https://twitter.com/soledadobrien/status/1222637252892942338	53
https://ushealthpharma.com/pelosiif-impeachments-fails-i-will-resign-immediately/	54
https://www.newsweek.com/trump-threatens-withdraw-aid-california-wildfires-1469873	55
https://thefederalist.com/2019/09/27/intel-community-secretly-gutted-requirement-of-first-hand-whistleblower-knowledge/	56
https://www.facebook.com/john.morano.96/posts/3413463322015404	57
https://www.newsweek.com/biden-says-over-150-million-americans-killed-gun-violence-since-2007-which-would-third-us-1489115	58
https://dailycaller.com/2019/12/01/biden-children-leg-hair/	59
https://twitter.com/KayaJones/status/1179224969642938368	60
https://www.facebook.com/AP.i.am.awake/photos/a.2273198979374324/3365236183503926/	61
https://www.facebook.com/RealTrumpNation/photos/a.744441295687017/1691648380966299/	62
https://obamawatcher.com/2019/10/george-was-obama-in-disguise/	63
https://newsmag.pro/celebrities-call-for-total-hollywood-strike-until-trump-resigns/	64
https://ktkmediagh.com/trump-announce-name-of-vaccine-to-cure-corona-virus-in-three-hours/	65
https://www.facebook.com/sandra.feno/posts/3869474993092657	66
https://halturnerradioshow.com/index.php/en/news-page/news-nation/virginia-governor-reportedly-orders-plans-to-cut-electric-phones-internet-for-gun-confiscations	67
https://www.facebook.com/AnonymousNewsNetwork/photos/a.790147877730457/2604897786255448/	68
https://www.facebook.com/three.percent.nation/photos/a.182961539037603/452063342127420/	69
https://massachusettsdaily.com/2020/01/06/nancy-pelosi-assures-iran-that-trump-will-pay-for-his-crimes/	70
https://thepoliticalbrief.com/	71
https://www.facebook.com/ben.hart.1042/posts/10157465624892705	72
https://conservativetears.com/2019/12/14/carol-burnette-dead-at-89-a-huge-trump-supporter-lost/	73

Table 4: Final sample of fact-checked items *(continued)*

URL	Item ID
https://archive.is/LQ7E5	74
https://www.facebook.com/three.percent.nation/photos/a.182961539037603/452063342127420/	75
Re-constructed from https://www.snopes.com/fact-check/coronavirus-doomsday-asteroid/	76
https://www.facebook.com/mickespn/posts/10216441379683361	77
https://www.facebook.com/photo.php	78
https://www.teaparty.org/huge-results-from-breaking-chloroquine-study-show-100-cure-rate-for-patients-infected-with-the-coronavirus-432559/	79
https://archive.is/KKPkd	80

B.5 Ideological valence pre-testing

Ideological valence of the 80 news items was pre-tested between June 19 and 30, 2020 with two different opt-in samples on Prolific and MTurk. To improve quality of raters, MTurk participants had to have voted in the 2016 presidential elections. Prolific raters also had to have indicated "politics" has a hobby, and were recruited so that there was an equal number of moderates, liberals and conservatives.

After giving consent and answering some sociodemographic questions, respondents were introduced to the task with the following text: "We would like you to look at about ten news reports/posts we have found online. For each of them, we would like you to assume that the information is completely accurate, and to answer the following questions: 1) Is this information more favorable to conservatives or liberals or neither group? 2) Is this information more consistent with the beliefs of conservatives, or of liberals, or of neither group? The answer options go from '-2 (liberals)' to '2 (conservatives)'. A good part of the reports/posts might not have such relevance for either side. In that case, do not hesitate to choose '0'!"

In the main task, each respondent saw a random subset of 10 out of the 80 news items. For each item, respondents were asked to respond to two questions: "Assume the above information is entirely accurate. Is it–or was it at the time of publishing–more favorable to liberals or conservatives or neither?" and "Assume the above information is entirely accurate. Is it–or was it at the time of publishing–more consistent with the beliefs of liberals or of conservatives or neither?", both with response scales from "-2 (liberals)" to "2 (conservatives)". After rating the ten items, some more covariates were measured and respondents were debriefed, i.e., received corrections about any false items they saw.

Figure 2 below shows that item-level average ratings are highly correlated both between measures and between samples. Table 5 lists all items along with their truth value, and the average valence rating (number of raters per item and standard error in parenthesis).

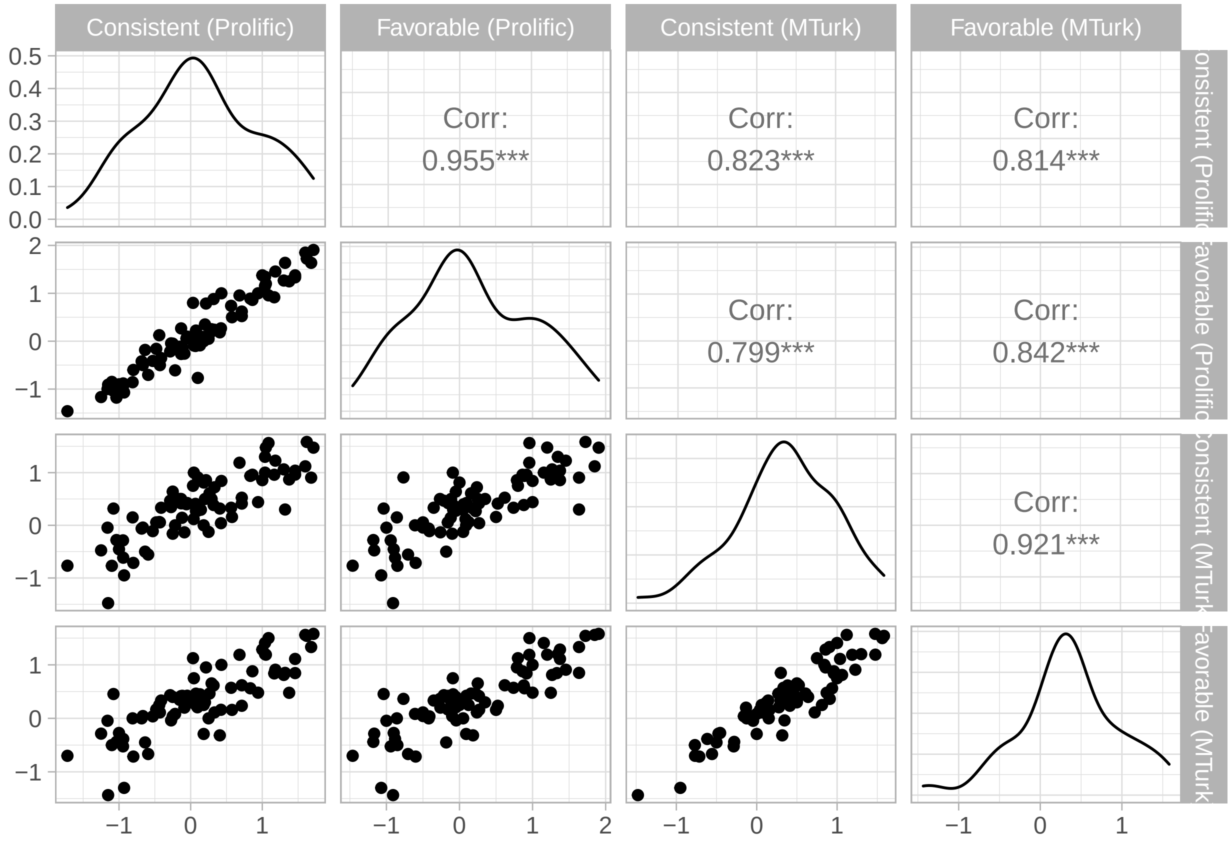

Figure 2: Pretest rating correlations

Table 5: Sample of news items: Content, truth, average valence (number of raters per item and standard error in parentheses)

Headline or first sentence	Item ID	Truth	Valence ‘consistent’	Valence ‘favorable’
Don Cheadle: ‘Another Trump Presidency Could End Us All’	1	true	-1.29 (n=56, SE=0.17)	-1.12 (n=56, SE=0.17)
Bulgaria coach sorry for racist chants, six people detained	2	true	-0.59 (n=39, SE=0.19)	-0.71 (n=38, SE=0.17)
Romney, Collins say Bolton report strengthens case for witnesses, makes them increasingly likely	3	true	-0.68 (n=53, SE=0.17)	-0.83 (n=53, SE=0.18)
Mexico’s state-run Pemex posts steep 2019 loss in blow to president’s revival plan	4	true	0.08 (n=36, SE=0.13)	0.03 (n=36, SE=0.13)
ACLU appeals in case alleging WV trans teen harassment	5	true	-0.64 (n=47, SE=0.20)	-0.55 (n=47, SE=0.20)
Mexico: More social spending, no big business bailout	6	true	-0.10 (n=38, SE=0.23)	-0.08 (n=38, SE=0.21)
The co-op board of an Upper West Side building turned away a doctor who came to NYC to treat coronavirus patients, and it highlights yet another ch...	7	true	-0.20 (n=45, SE=0.15)	-0.02 (n=45, SE=0.15)
UK on course for ’significant’ recession after March slump	8	true	0.20 (n=50, SE=0.13)	0.10 (n=49, SE=0.13)
U. of Wisconsin-Madison Furloughs Employees Despite Accepting $10 Million in Coronavirus Relief	9	true	0.35 (n=40, SE=0.18)	0.32 (n=40, SE=0.19)
Dozens of countries are testing an NSO platform to track citizens infected with coronavirus. Here’s a sneak peak at how it works.	10	true	0.09 (n=46, SE=0.14)	-0.11 (n=46, SE=0.13)
UConn to propose increased tuition for all students	11	true	0.17 (n=52, SE=0.13)	0.12 (n=52, SE=0.12)
WHO anti-cholera vaccination campaign begins in Sudan	12	true	-0.26 (n=43, SE=0.16)	-0.02 (n=43, SE=0.14)
Man admits attempted arson at Planned Parenthood clinic	13	true	0.44 (n=52, SE=0.18)	-0.29 (n=52, SE=0.19)

Table 5: Sample of news items: Content, truth, average valence (number of raters per item and standard error in parentheses) *(continued)*

Headline or first sentence	Item ID	Truth	Valence ‘consistent’	Valence ‘favorable’
Fauci: ’We’re not there yet’ on key steps to reopen economy	14	true	-0.91 (n=46, SE=0.18)	-0.65 (n=46, SE=0.19)
Switzerland might increase 20 billion franc emergency scheme - Finance Minister	15	true	0.06 (n=47, SE=0.19)	0.09 (n=47, SE=0.17)
Former acting AG calls Whitaker says upcoming IG report ’most consequential’ in many years	16	true	0.17 (n=53, SE=0.21)	0.34 (n=53, SE=0.21)
White House trade adviser touts new jobs report: Trump is ’focused on job creation every day’	17	true	1.30 (n=43, SE=0.15)	1.49 (n=43, SE=0.14)
Australia defends choice of remote detention center to house locals evacuated from Wuhan	18	true	0.51 (n=39, SE=0.16)	0.18 (n=39, SE=0.16)
Explosion erupts at Texas chemical refinery	19	true	-0.16 (n=32, SE=0.14)	-0.16 (n=32, SE=0.17)
Nuclear command nominee sidesteps questions on arms control treaties	20	true	0.23 (n=57, SE=0.16)	0.07 (n=57, SE=0.15)
Cambodian opposition leader meets ambassador after house arrest lifted	21	true	0.19 (n=37, SE=0.16)	0.18 (n=38, SE=0.14)
Family of Wichita man killed by police in swatting incident seeking $25 million from city	22	true	-0.71 (n=42, SE=0.16)	-0.57 (n=42, SE=0.15)
Australia Foreign Minister says helping White House probe in national interest	23	true	0.26 (n=51, SE=0.18)	0.37 (n=51, SE=0.19)
Indonesia reports 496 new coronavirus infections, 43 deaths	24	true	0.11 (n=45, SE=0.12)	0.09 (n=45, SE=0.12)
Mom wants answers after girl taken to mental health center	25	true	-0.40 (n=35, SE=0.18)	-0.23 (n=35, SE=0.19)
Donald Trump Jr. Accuses Democrats of Using ’Gestapo Tactics,’ Calls Impeachment Proceedings ’Kangaroo Court Stuff’	26	true	1.20 (n=50, SE=0.17)	1.08 (n=50, SE=0.18)

Table 5: Sample of news items: Content, truth, average valence (number of raters per item and standard error in parentheses) *(continued)*

Headline or first sentence	Item ID	Truth	Valence 'consistent'	Valence 'favorable'
More than 2 dozen mumps cases seen at University of Arkansas	27	true	0.00 (n=46, SE=0.11)	0.09 (n=46, SE=0.12)
Coroner: Man found in vacant house died of gunshot wounds	28	true	0.24 (n=37, SE=0.12)	0.11 (n=37, SE=0.12)
Wealthy cities have more coronavirus cases — but, in New York, poorer communities are more affected	29	true	-0.36 (n=47, SE=0.16)	-0.23 (n=47, SE=0.15)
Data on arthritis drug to treat coronavirus could come within weeks: Regeneron executive	30	true	0.40 (n=50, SE=0.12)	0.46 (n=50, SE=0.12)
Harris, Waters release $100 billion affordable housing bill	31	true	-1.20 (n=55, SE=0.17)	-1.05 (n=56, SE=0.16)
State places consumption limits on fish caught in Squam Lake	32	true	-0.57 (n=42, SE=0.16)	-0.31 (n=42, SE=0.17)
Will Gilead price its coronavirus drug for public good or company profit?	33	true	0.06 (n=48, SE=0.16)	-0.04 (n=48, SE=0.14)
White House reporter tests negative for coronavirus	34	true	0.23 (n=44, SE=0.15)	0.23 (n=44, SE=0.13)
Brexit deal done - two EU officials	35	true	0.88 (n=34, SE=0.16)	0.74 (n=34, SE=0.18)
Security service employee dead in Moscow shooting: Interfax cites FSB	36	true	0.23 (n=48, SE=0.11)	0.21 (n=48, SE=0.12)
Thousands of U.S. banks may sit out small-business rescue plan on liability worries: sources	37	true	0.07 (n=41, SE=0.15)	0.17 (n=41, SE=0.14)
Vintage WWII fighter plane to fly sightseers over Hawaii	38	true	0.47 (n=47, SE=0.12)	0.34 (n=47, SE=0.13)
Schools shut in Lahore as city chokes in toxic smog	39	true	-0.76 (n=34, SE=0.18)	-0.65 (n=34, SE=0.17)
Hundreds urge lawmakers to keep religious vaccine exemption	40	true	0.45 (n=38, SE=0.22)	0.10 (n=38, SE=0.21)

Table 5: Sample of news items: Content, truth, average valence (number of raters per item and standard error in parentheses) *(continued)*

Headline or first sentence	Item ID	Truth	Valence ‘consistent’	Valence ‘favorable’
ICE Fails To Properly Redact Document, Reveals Location Of Future ’Urban Warfare’ Training Facility	41	true	-0.58 (n=45, SE=0.17)	-0.69 (n=45, SE=0.18)
Bernie Sanders said at the debate last night he wants minimum wage to be $15 an hour. 15$ x 40 hr work week = $600 600$ x 52 weeks per year = $...	42	false	0.46 (n=44, SE=0.25)	0.66 (n=44, SE=0.23)
Boris Johnson ’hides in a fridge’ to avoid Piers Morgan interview	43	true	-0.34 (n=41, SE=0.21)	-0.44 (n=41, SE=0.22)
Nicole Kidman ’blacklisted’ from Hollywood for vowing to support president	44	false	0.62 (n=40, SE=0.26)	1.00 (n=40, SE=0.19)
The UN Plans To Implant Everyone With A Biometric ID	45	false	0.60 (n=35, SE=0.26)	0.89 (n=35, SE=0.22)
Judge Rules High School Girls MUST Shower With Boys, says Girls Have No Right To Privacy	46	false	0.45 (n=38, SE=0.25)	0.26 (n=38, SE=0.26)
Doctor warned US hospitals should prepare for as many as 96 MILLION coronavirus infections and nearly HALF A MILLION deaths	47	false	-0.89 (n=45, SE=0.18)	-0.76 (n=45, SE=0.17)
Video Resurfaces of Michelle Obama Praising ’Wonderful Human Being’ Harvey Weinstein After Sex Abuse Conviction	48	true	1.15 (n=46, SE=0.19)	1.28 (n=46, SE=0.18)
ACTOR WILL SMITH: ’I’M NOT AFRAID TO SAY THAT TRUMP AND HIS SUPPORTERS ARE Ba...	49	false	-0.74 (n=43, SE=0.21)	-0.58 (n=43, SE=0.23)
A Missouri bill would cut off aid to libraries that allow kids to access ’age-inappropriate sexual materials’	50	true	0.62 (n=42, SE=0.20)	0.62 (n=42, SE=0.20)
Bloomberg spent $500 million on ads. The U.S. population is 327 million. He could have given each American $1 million and still have money left ove...	51	false	-0.19 (n=32, SE=0.23)	0.19 (n=32, SE=0.25)
Joe Biden said: People who have never died before are now dying from coronavirus.	52	false	0.34 (n=38, SE=0.21)	0.79 (n=38, SE=0.21)

Table 5: Sample of news items: Content, truth, average valence (number of raters per item and standard error in parentheses) *(continued)*

Headline or first sentence	Item ID	Truth	Valence 'consistent'	Valence 'favorable'
Trump's 2020 budget includes $25,000,000,000 in cuts to Social Security.	53	true	0.37 (n=49, SE=0.23)	-0.04 (n=49, SE=0.25)
Pelosi:'If Impeachment Fails, I Will Resign Immediately'	54	false	1.27 (n=41, SE=0.20)	1.20 (n=41, SE=0.20)
Russia Says Trump Once Offered to Help Putin Fight Wildfires—Now He's Threatening to Withdraw Aid From California	55	true	-0.94 (n=48, SE=0.18)	-1.17 (n=48, SE=0.16)
Intel Community Secretly Gutted Requirement Of First-Hand Whistleblower Knowledge	56	false	-0.27 (n=44, SE=0.23)	-0.14 (n=44, SE=0.23)
Staged Event? These officers were involved with something, I'm not sure exactly what, but something is just not adding up. I think there is at the ...	57	false	0.33 (n=64, SE=0.19)	0.30 (n=64, SE=0.19)
Biden Says Over 150 Million Americans Killed by Gun Violence Since 2007, Which Would Be Half of U.S. Population	58	true	0.35 (n=54, SE=0.20)	0.94 (n=54, SE=0.17)
Biden Described Allowing Children To Play With His Wet Leg Hair In Resurfaced Clip: 'I've Loved Kids Jumping On My Lap'	59	true	0.83 (n=42, SE=0.19)	1.26 (n=42, SE=0.15)
Did you know that if the impeachment reaches the house and passes to the senate and isn't passed the first term is nullified and Trump can run two ...	60	false	1.21 (n=44, SE=0.16)	1.18 (n=44, SE=0.18)
Anthony Fauci stands to lose 100 million dollars on Bill gates 'vaccine' for the coronavirus. Because he invested in this vaccine. If ever...	61	false	1.06 (n=49, SE=0.16)	0.88 (n=49, SE=0.17)
Barack Hussien Obama's real name is: Barry Soetoro. Kamala Harris' real name is: Maya Harrison. Ilha Omar real name is: Ilhan Esmi...	62	false	1.24 (n=51, SE=0.14)	1.24 (n=51, SE=0.14)

Table 5: Sample of news items: Content, truth, average valence (number of raters per item and standard error in parentheses) *(continued)*

Headline or first sentence	Item ID	Truth	Valence 'consistent'	Valence 'favorable'
'He's Low Risk,' Said Obama Before Freeing ISIS Leader Al Baghdadi	63	false	1.60 (n=40, SE=0.12)	1.75 (n=40, SE=0.10)
CELEBRITIES CALL FOR 'TOTAL HOLLYWOOD STRIKE' UNTIL TRUMP RESIGNS	64	false	-0.44 (n=48, SE=0.24)	-0.35 (n=48, SE=0.25)
Trump Announce Name Of Vaccine To Cure Corona Virus In Three Hours	65	false	0.93 (n=45, SE=0.17)	1.33 (n=45, SE=0.18)
I just want to share what Goodwill Industries just did to all their employees. They use this pandemic to fire all their employees instead of laying...	66	false	-0.11 (n=47, SE=0.20)	-0.26 (n=47, SE=0.18)
VIRGINIA GOVERNOR REPORTEDLY ORDERS PLANS TO CUT ELECTRIC, PHONES, INTERNET FOR GUN CONFISCATIONS!	67	false	0.21 (n=47, SE=0.25)	0.28 (n=47, SE=0.24)
Do you know what really happens when you donate your birthday to charity on Facebook?	68	false	0.21 (n=43, SE=0.14)	0.16 (n=43, SE=0.12)
In 1988 Iran nearly sank the USS Samuel B. Roberts. In response Reagan destroyed half of Iran's navy.	69	true	1.28 (n=39, SE=0.14)	1.18 (n=39, SE=0.15)
Nancy Pelosi Assures Iran That Trump Will 'Pay For His Crimes'	70	false	0.98 (n=54, SE=0.19)	1.09 (n=54, SE=0.18)
Biden Caught In Damaging Gaffe On Hot Mic – Tells Men To Marry Into Families 'With 3 Or 4 Sisters'	71	false	1.33 (n=45, SE=0.16)	1.69 (n=45, SE=0.10)
Do you wonder why CA only has 559 CV19 deaths compared to 7,067 deaths in NY? Welp, researchers at Stanford's School of Medicine suspect it...	72	false	1.18 (n=62, SE=0.15)	1.03 (n=62, SE=0.13)
Carol Burnette Dead at 89 – A Huge Trump Supporter Lost	73	false	1.08 (n=39, SE=0.19)	0.80 (n=39, SE=0.20)
Supreme Court Ends Pelosi's Reign Of Terror With Landmark Decision On Impeachment	74	false	1.60 (n=53, SE=0.11)	1.64 (n=53, SE=0.12)

Table 5: Sample of news items: Content, truth, average valence (number of raters per item and standard error in parentheses) *(continued)*

Headline or first sentence	Item ID	Truth	Valence 'consistent'	Valence 'favorable'
Churchgoers Must Register With the Government in Kansas City	75	false	0.91 (n=54, SE=0.18)	0.87 (n=54, SE=0.18)
The World Deserves the Truth.... Please brace yourself for the following information I'm about to share with you. It's not going to be easy to unders...	76	false	0.58 (n=38, SE=0.17)	0.40 (n=38, SE=0.16)
Check this out! Hospitals get $750 if you die from the flu, and $17,500 if you died from COVID-19. Now think about that!	77	false	0.64 (n=42, SE=0.19)	0.69 (n=42, SE=0.17)
SERIOUS EXCELLENT ADVICE by Japanese doctors treating COVID-19 cases. Everyone should ensure your mouth & throat is moist, never DRY. Take a few si...	78	false	0.23 (n=51, SE=0.13)	0.22 (n=51, SE=0.14)
Huge! Results From Breaking Chloroquine Study Show 100percent Cure Rate For Patients Infected With The Coronavirus	79	false	1.02 (n=48, SE=0.15)	1.27 (n=48, SE=0.12)
BREAKING: All Big 3 networks ABC, CBS, NBC blacked out Trump defense lawyer Pam Bondi's presentation this afternoon of Ukraine corruption related t...	80	false	0.24 (n=45, SE=0.25)	0.20 (n=45, SE=0.26)

C Subject sample

Tables 6 through 9 compares the sample with population statistics on basic sociodemographic variables.

Table 6: Gender distribution of population and sample

		Sample	
Sex	Population percent[a]	Abs.	Percent
Male	49	678	48.71
Female	51	714	51.29

[a] Source: U.S. Census Bureau, Current Population Survey, 2018 Annual Social and Economic Supplement, https://www.census.gov/data/tables/2018

Table 7: Age distribution of population and sample

		Sample	
Age group	Population percent[a]	Abs.	Percent
18-25	11.77	165	11.84
26-34	16.23	220	15.79
35-54	34.17	478	34.31
55-64	17.29	242	17.37
65+	20.54	288	20.67

[a] Source: U.S. Census Bureau, Current Population Survey, 2018 Annual Social and Economic Supplement, https://www.census.gov/data/tables/2018

Table 8: Education distribution of population and sample

		Sample	
Age group	Population percent[a]	Abs.	Percent
18-25	11.77	165	11.84
26-34	16.23	220	15.79
35-54	34.17	478	34.31
55-64	17.29	242	17.37
65+	20.54	288	20.67

[a] Source: U.S. Census Bureau, Current Population Survey, 2018 Annual Social and Economic Supplement, https://www.census.gov/data/tables/2018

Table 9: Partisan distribution of population and sample

		Sample	
Partisanship	Population percent[a]	Abs.	Percent
Democrat	33	482	34.60
Republican	26	373	26.78

Non-partisan	37	538	38.62

[a] Source: Pew Research Center 2018, https://www.people-press.org/2018/03/20/party-identification-trends-1992-2017/

D Variable measurement

Variable	Question	Choices/Coding
Age	"How old are you?"	Number
Gender	"What is your gender?"	"Male", "Female", Other"
Education	"What is the highest level of school you have completed or the highest degree you have received?"	5 choices from "12th grade without diploma or less" to "Master's degree, professional school degree, or doctorate degree"
Partisanship	"Generally speaking, do you usually think of yourself as a Democrat, a Republican, an Independent, or what?"	"Democrat", "Republican", "Independent", "Other party", "No preference"
Ideology	"Here is a seven-point scale on which the political views that people might hold are arranged from extremely liberal to extremely conservative. Where would you place yourself on this scale?	7-point scale from "Extremely liberal" to "Extremely conservative" and "Haven't thought much about this"
Turnout	"In 2016 Hillary Clinton ran on the Democratic ticket against Donald Trump who ran for the Republicans. Do you remember for sure whether or not you voted in that election?"	"Yes, voted", "No, didn't vote"
Facebook use	"How often do you use Facebook?"	9-point scale from "Many times every day" to "Never" and "I don't have an account"
General media trust	"In general, how much trust and confidence do you have in the mass media – such as newspapers, TV and radio – when it comes to reporting the news fully, accurately, and fairly?"	7-point scale from "0 - none at all" to "6 - a great deal"
Digital literacy	"How familiar are you with the following computer and Internet-related items? Please choose a number between 0 and 6 where 0 represents 'no understanding' and 5 represents 'full understanding' of the item."	Separate 7-point scales for "Pishing", "Hashtag", "JPG", "Malware", "Cache", "RSS"

Variable	Question	Choices/Coding
Economic left-right item 1	“Some people think the government should provide fewer services even in areas such as health and education in order to reduce spending, others feel it is important for the government to provide many more services even if it means an increase in spending. Where would you place yourself on a scale from 1 to 7?”	7-point scale from “0 - Government should provide fewer services” to “6 - Government should provide more services”
Economic left-right item 2	“Some people feel the government in Washington should see to it that every person has a job and a good standard of living. Others think the government should just let each person get ahead on their own. Where would you place yourself on a scale from 1 to 7?”	7-point scale from “0 - Government should let people get ahead on their own” to “6 - Government should see to jobs and living standards”
Social left-right item 2	“There has been some discussion about abortion during recent years. Which one of the opinions below best agrees with your view?”	4 choices from “By law, abortion should never be permitted” to “By law, a woman should always be able to obtain an abortion as a matter of personal choice.”
Social left-right item 2	Do you think gay or lesbian couples should be legally permitted to adopt children?	“Yes”, “No”
Social left-right item 3	“Do you think it is better, worse, or makes no difference for the family as a whole if the man works outside the home and the woman takes care of the home and family?”	“Worse”, “Better”, “Makes no difference”
Need for closure item 1	“How disorganized are the rooms that you personally live and work in most?”	7-point scale from “0 - not disorganized at all” to “7 - Extremely disorganized”
Need for closure item 2	“Do you like unpredictable situations or dislike them?”	7-point scale from “0 - dislike unpredictable situations” to “7 - extremely disorganized”
Need for closure item 3	“How many of your important decisions do you make quickly and confidently?”	7-point scale from “0 - None” to “6 - All”
Need for closure item 4	“When you don’t understand the reason why something happens in your life, how uncomfortable does that make you feel?”	7-point scale from “0 - Not uncomfortable at all” to “6 - Very uncomfortable”

Variable	Question	Choices/Coding
Need for closure item 5	"In the situations when you see two people in a conflict with one another, how often can you see how both sides could be right?"	7-point scale from "0 - Always" to "6 - Never"
Cognitive reflection item 1	"A bat and a ball cost $1.10 in total. The bat costs $1.00 more than the ball. How much does the ball cost?"	Open-ended
Cognitive reflection item 2	"In a lake, there is a patch of lily pads. Every day, the patch doubles in size. If it takes 48 days for the patch to cover the entire lake, how long would it take for the patch to cover half of the lake?"	Open-ended
Cognitive reflection item 3	"Emily's father has three daughters. The first two arenamed April and May. What is the third daughter's name?"	Open-ended
Cognitive reflection item 4	"If you're running a race and you pass the person in second place, what place are you in?"	Open-ended
Ethnicity	"Here is a list of five race categories. Please choose one or more races that you consider yourself to be"	"White"," Black or African-American", "American Indian or Alaska Native", "Asian", "Native Hawaiian or other Pacific Islander"
State of residence	"In what U.S. state do you live?"	Choice among 50 federal states and "DC"
Income	"What was your total income in 2019? This figure should include income from all sources, including salaries, wages, pensions, Social Security, dividends, interest, and all other income"	Open-ended

E Differences to pre-registration

Research questions

- Renamed RQ1a from the pre-analysis plan to RQ1; RQ2a to RQ2; RQ2b to RQ3.

Measurement

- Age measured with question “How old are you?” instead of “What is the year of your birth?”
- Gender third option “Other” instead of “Other/Don’t want to say”
- State of residence measured with question “In what U.S. state do you live?” instead of “In what state are you registered to vote now?”
- Partisanship measured with question “Generally speaking, do you usually think of yourself as a Democrat, a Republican, an Independent, or what?” instead of “What political party are you registered with, if any?”, and only three options.
- Length of media use per day (“On average, how many minutes per day would you say you spent reading, watching, or listening to news about politics?”) and relevance of sources (“Which of those sources are your most important sources of news?”) was not measured.
- Digital literacy measured on 7-point instead of 5-point scales.
- Third item of economic left-right battery (“We would like you to tell us whether you would like to see spending increased or decreased on aid to the poor”) was dropped.
- Need for closure items were measured on end-labelled seven-point scales instead of fully labelled five-point scale
- The outcome (belief) was measured with the question “To the best of your knowledge, how accurate is the information in the above item? 0 means not at all, 6 means completely.” from Pennycook and Rand (2020) instead of the question “Do you believe that this information is true? 0 means not at all, 6 means completely.”

Analysis

- Instead of clustering standard errors only at the subject level, I also clustered them at the item level. This does not change estimates, but created more conservative standard errors.

F Ideological asymmetries and covariates

F.1 Correlations of ideology and covariates

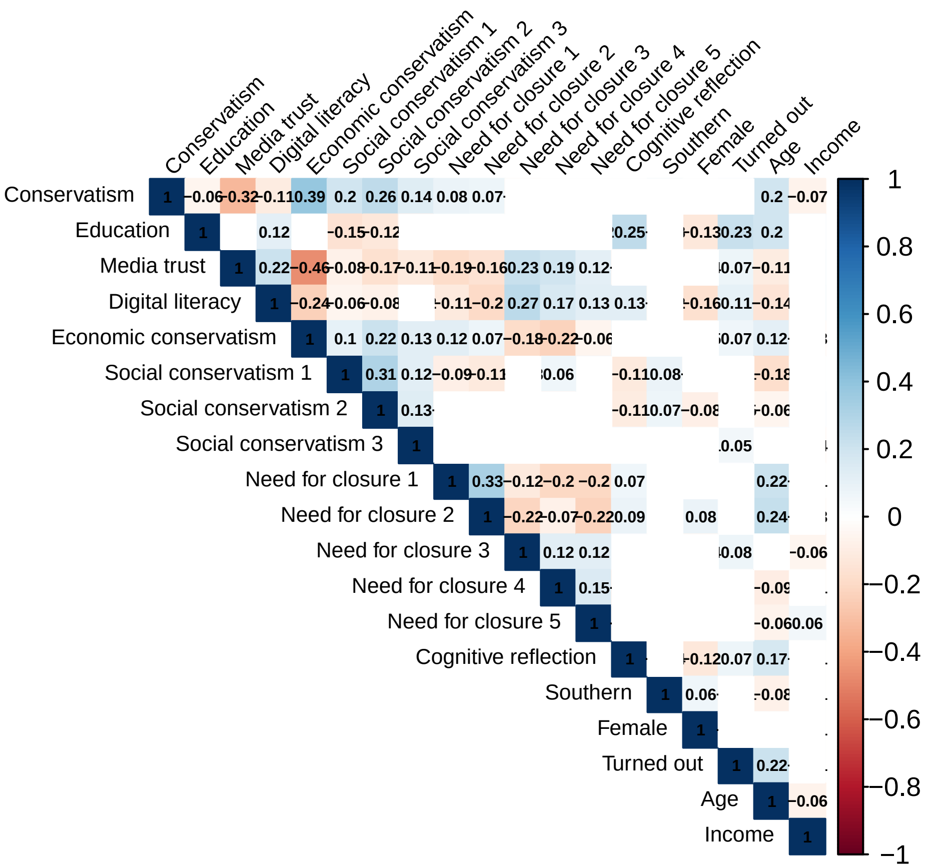

Figure 3: Covariates of individual ideology

F.2 Regressions with covariates

The regression below include interactions with some of the variables shown in Figure 3 above. The left columns of tables 11 and 12 shows the same models as reported in the main text. The middle columns include interactions of the truth or congruence of the news item with all variables correlated to ideology. The rightmost column includes only variables with a significant interaction term, and serves as the basis for the marginal-effects plots. In sum, it shows that none of the measured variables can explain away the ideological asymmetries found. But some variables reveal interesting interactions in themselves: For example, higher age, higher digital literacy and greater media trust all contribute positively to truth discernment, as seen in Figure 4.

Table 11: Truth discernment regression, with covariates

	Belief		
	RQ1	RQ1 with all covariates	RQ1 with significant covariates
Ideology	0.04 (0.03)	0.10*** (0.03)	0.11*** (0.03)
Digital literacy		0.02 (0.03)	0.05 (0.03)
Age		−0.02*** (0.003)	−0.02*** (0.003)
Education		−0.07 (0.04)	−0.08* (0.04)
Media trust		0.11** (0.03)	0.16*** (0.03)
Econ. conservatism (index)		−0.02 (0.04)	
Soc. conservatism 1		0.13** (0.05)	
Soc. conservatism 2		0.34*** (0.10)	0.41*** (0.09)
Soc. conservatism 3		−0.01 (0.07)	
Need for closure 1		−0.06* (0.03)	
Need for closure 2		−0.16*** (0.03)	−0.20*** (0.03)
Need for closure 3		0.07 (0.04)	
Need for closure 4		0.11** (0.03)	
Need for closure 5		0.05 (0.04)	
Item truth	0.92*** (0.11)	−1.03* (0.40)	−1.47*** (0.29)
Truth * Ideology	−0.10** (0.04)	−0.08* (0.03)	−0.09* (0.04)
Truth * Digital literacy		0.12*** (0.03)	0.11*** (0.03)
Truth * Age		0.02*** (0.003)	0.02*** (0.004)
Truth * Education		0.11* (0.04)	0.12** (0.04)
Truth * Media trust		0.08* (0.04)	0.07* (0.03)
Truth * Economic conservatism		−0.03 (0.04)	
Truth * Soc. conservatism 1		−0.09 (0.05)	
Truth * Soc. conservatism 2		−0.31** (0.11)	−0.40*** (0.10)
Truth * Soc. conservatism 3		0.04 (0.07)	
Truth * Need for closure 1		0.06 (0.03)	
Truth * Need for closure 2		0.12*** (0.04)	0.16*** (0.03)
Truth * Need for closure 3		−0.03 (0.04)	
Truth * Need for closure 4		−0.02 (0.03)	
Truth * Need for closure 5		−0.06 (0.04)	
Constant	2.55*** (0.09)	3.09*** (0.38)	3.77*** (0.27)
Observations	10,431	9,754	10,016
R^2	0.06	0.17	0.16

Note: *p<0.05; **p<0.01; ***p<0.001

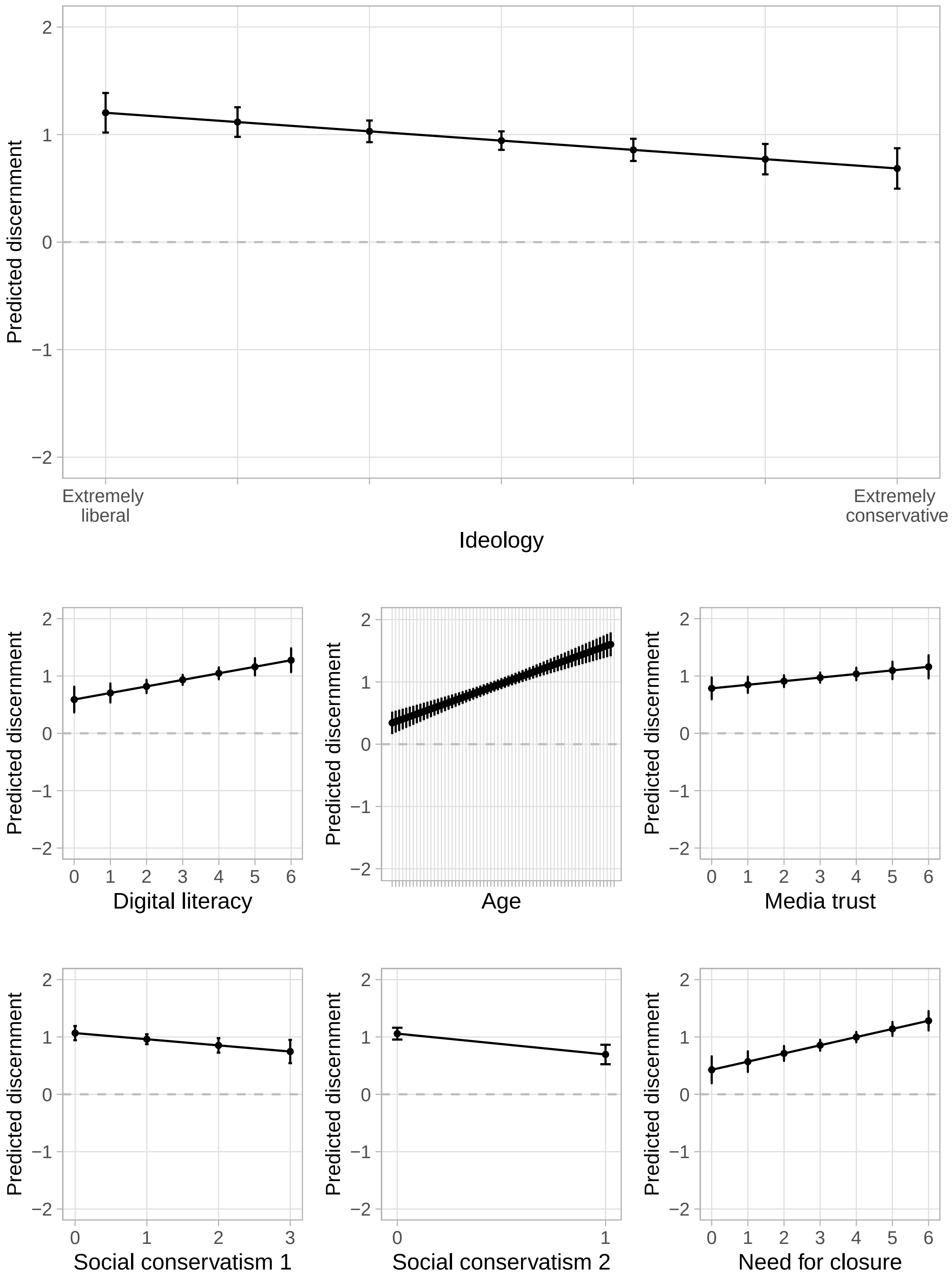

Figure 4: Marginal effects of RQ1 regression with covariates

Table 12: Bias regression, with covariates

	Belief		
	RQ2	RQ2 with all covariates	RQ2 with significant covariates
Ideology	0.04 (0.04)	0.13*** (0.03)	0.07* (0.04)
Digital literacy		0.08* (0.03)	
Age		−0.01* (0.003)	−0.01*** (0.003)
Education		−0.04 (0.04)	
Media trust		0.18*** (0.03)	
Econ. conservatism (index)		−0.04 (0.04)	
Soc. conservatism 1		0.12* (0.05)	
Soc. conservatism 2		0.14 (0.11)	
Soc. conservatism 3		0.01 (0.07)	
Need for closure 1		−0.03 (0.03)	
Need for closure 2		−0.14*** (0.03)	−0.19*** (0.03)
Need for closure 3		0.04 (0.03)	
Need for closure 4		0.13*** (0.03)	
Need for closure 5		−0.03 (0.03)	0.03 (0.04)
Item truth	0.17** (0.06)	−0.80 (0.49)	−0.71** (0.27)
Truth * Ideology	−0.15* (0.06)	−0.20*** (0.05)	−0.17** (0.06)
Truth * Digital literacy		0.03 (0.04)	
Truth * Age		0.01* (0.003)	0.01 (0.003)
Truth * Education		0.02 (0.05)	
Truth * Media trust		−0.004 (0.04)	
Truth * Economic conservatism		0.03 (0.05)	
Truth * Soc. conservatism 1		−0.06 (0.06)	
Truth * Soc. conservatism 2		−0.02 (0.13)	
Truth * Soc. conservatism 3		−0.004 (0.08)	
Truth * Need for closure 1		0.02 (0.03)	
Truth * Need for closure 2		0.09** (0.03)	0.09* (0.04)
Truth * Need for closure 3		0.003 (0.04)	
Truth * Need for closure 4		−0.07 (0.04)	
Truth * Need for closure 5		0.11* (0.05)	0.08 (0.05)
Constant	3.14*** (0.08)	2.86*** (0.36)	4.33*** (0.23)
Observations	7,234	6,764	7,226
R^2	0.01	0.11	0.05
Note:			*p<0.05; **p<0.01; ***p<0.001

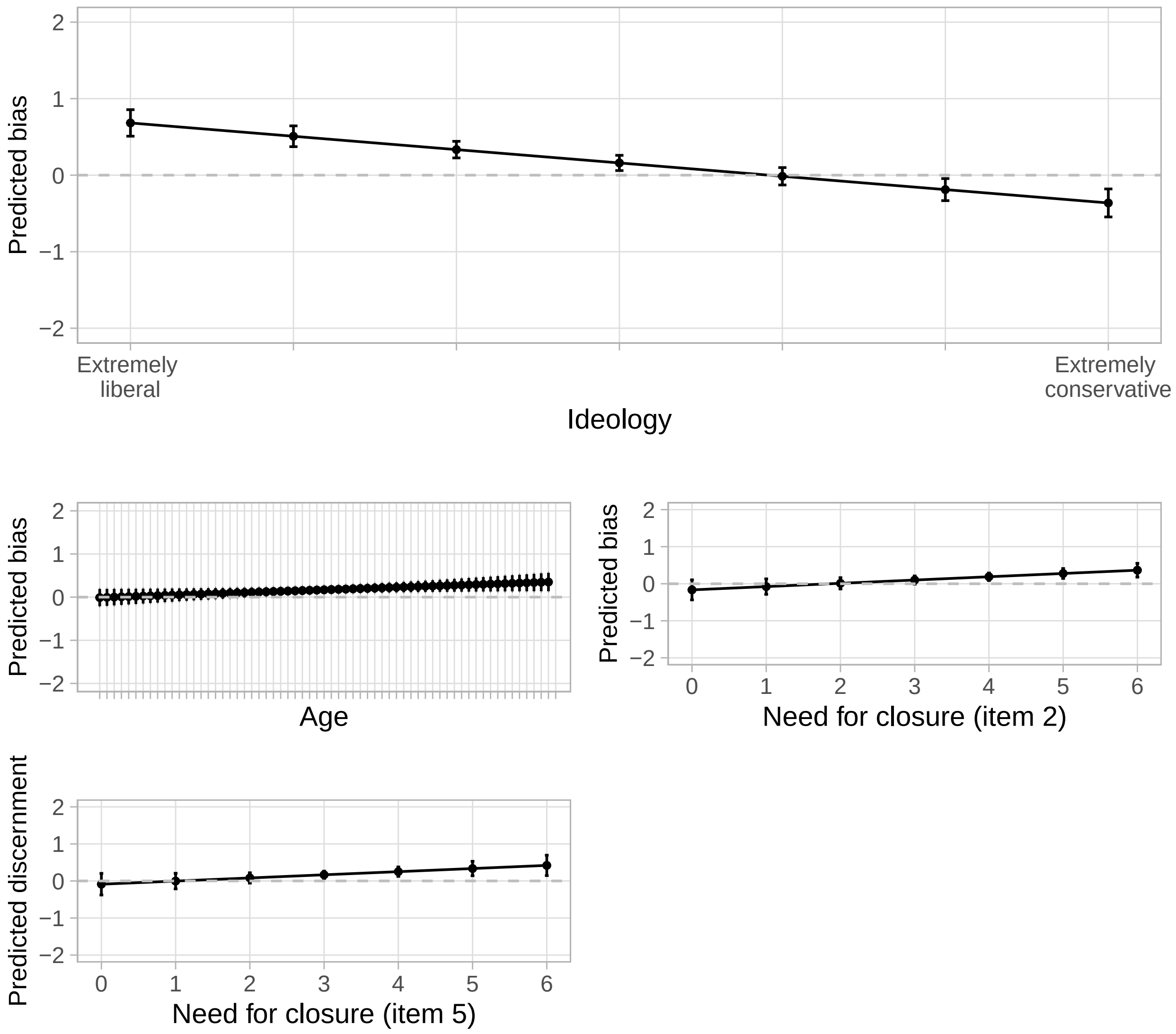

Figure 5: Marginal effects of RQ2 regression with covariates

G Plot of truth discernment-bias interaction (RQ3)

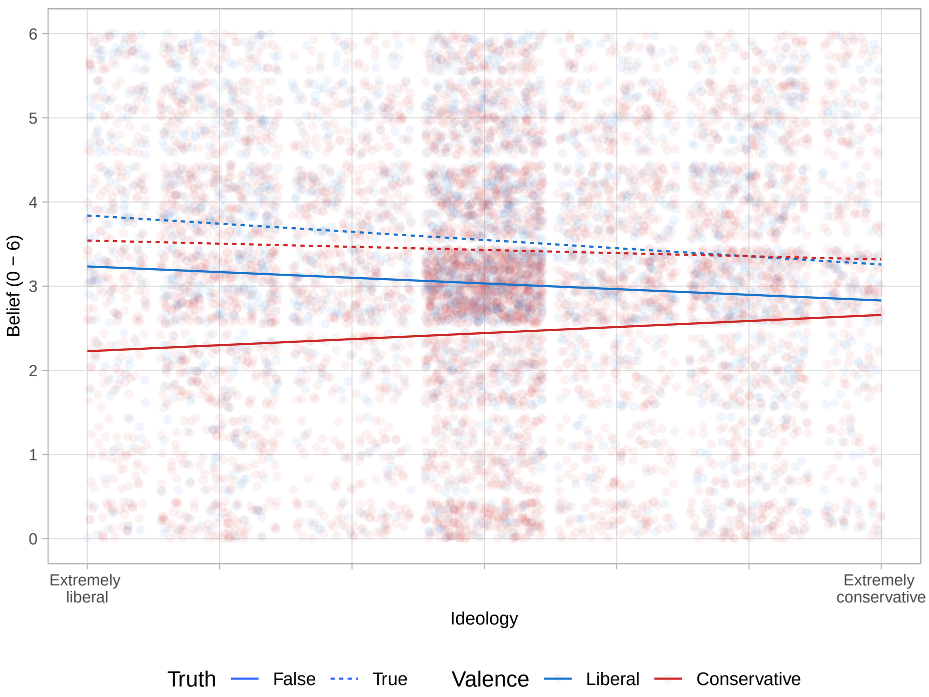

Figure 6: Truth discernment-bias interaction

H Re-estimating truth discernment with a balanced item sample

Instead of balancing the selection of items on some variable deemed relevant ex ante, as many previous studies have done, this study's design attempted to represent the true distribution of such variables in the population by randomly sampling from a larger collection. This also allows to balance on any measured item-level variable ex-post to get estimates similar to studies like Pennycook and Rand (2019). Specifically, such studies commonly select their news stimuli ensuring that there is an equal number (and degree) of false news that are in line with a conservative, and those that are in line with a liberal worldview. Thus, I re-estimate the model for truth discernment, as well as for bias, while varying ideological valence within the news item sample.

H.1 Truth discernment

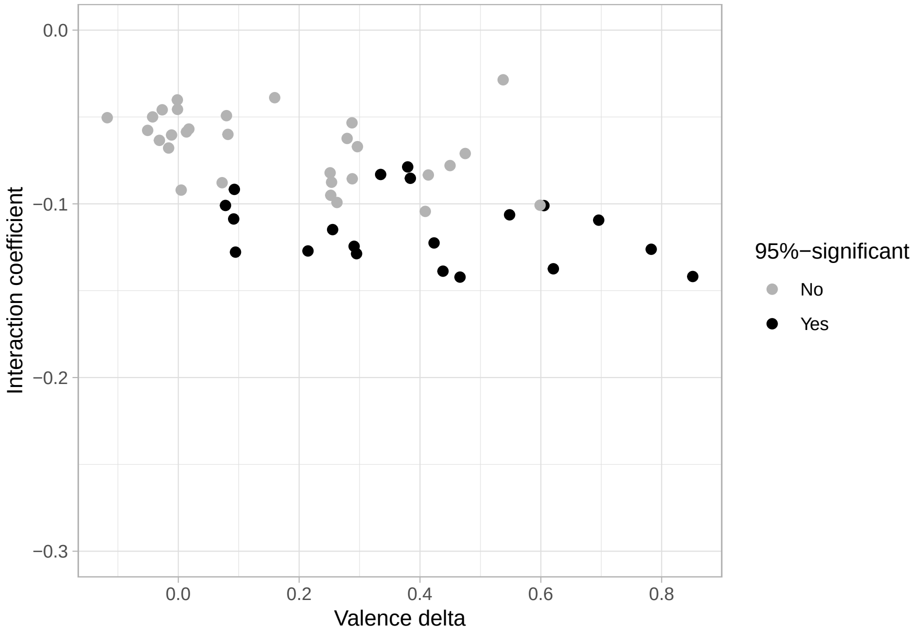

Figure 7: Effect of controlling for valence delta on truth discernment asymmetry

As shown in Figure 2 in the main paper, false items have a valence score that is slightly higher, i.e. more conservative, than true items. I refer to this difference as "valence delta". To understand how results depend more generally on the valence delta, I proceed as follows: Out of the complete sample of 80 items, I sample 20 true and 20 false items 50 times. To vary the extent to which false and true items differ in ideological valence across samples, I set different constraints while sampling: 10 samples are constrained to have a valence delta smaller or equal to zero; 10 samples a delta smaller than 0.1; further 10 a delta smaller than 0.3; further 10 a delta smaller than 0.5; and 10 are sampled with no constraint. For each of these samples, I run the same cluster-robust regression and record the relevant coefficients each time.

Figure 7 shows the truth-ideology interaction coefficient from all these regressions, plotted against the valence delta. Significant cases are colored in dark. As previously, a negative interaction coefficient means that conservatives are less truth discerning. It can be seen that with a small valence delta, the ideological asymmetry indeed decreases, which suggests that supply does matter. Overall, the correlation between valence delta and the regression coefficient is -0.56. However, among the samples with a valence delta around zero, a good part still reveals an asymmetry. These alternative estimates with a valence data of zero are more comparable with studies like Pennycook and Rand (2019). On the one hand, given that imperfectness of my data collection, there may be value in such balancing. On the other hand, it is still unclear what kind of population of information such balanced sub-samples represent. What is more, there are as many ways to conduct such balancing as there are many item-level variables potentially relevant. For example, familiarity with information has been shown to strongly influence belief (Brashier and Marsh 2020).

H.2 Bias

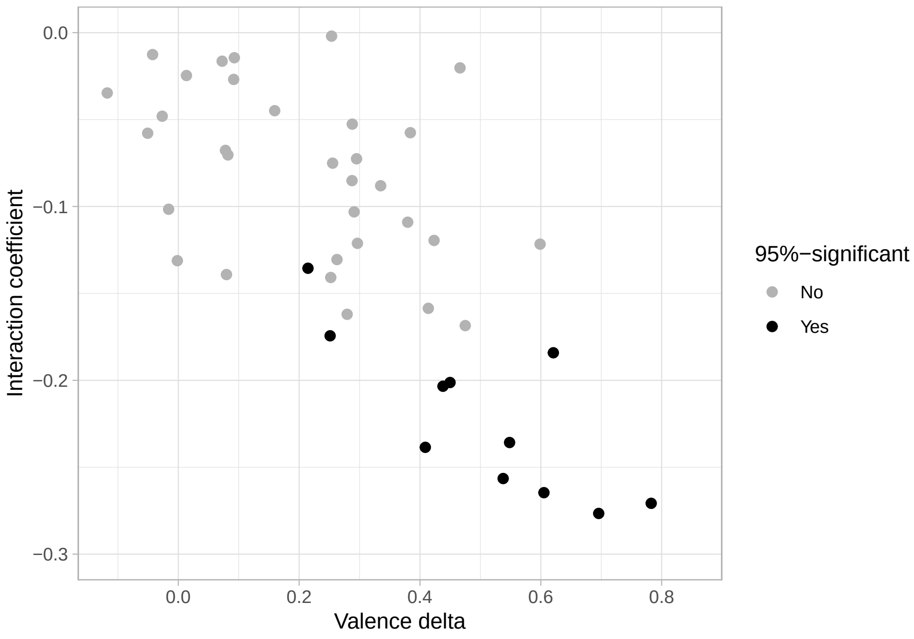

Figure 8: Effect of controlling for valence delta on bias asymmetry

To explore how balancing the sample affects asymmetries in bias, I apply the sample re-sampling strategy as in the previous subsection. For each of these samples, I run the same cluster-robust regression and record the relevant coefficients each time. Figure 8 shows the truth-ideology interaction coefficient from all these regressions, plotted against the valence delta. Significant cases are colored in dark. A negative interaction coefficient means that conservatives are less biased. It can be seen that with a small valence delta, the ideological asymmetry indeed decreases,. Overall, the correlation between valence delta and the regression coefficient is -0.79.

I Robustness checks

I.1 Continuous congruence variable

Table 13 shows results for RQ2 and RQ3 using a continuous version of the congruence variable, instead of the categorical version.

Table 13: Bias: continuous congruence

	Belief	
	RQ2	RQ3
Ideology	-0.04^{*}	0.003
	(0.02)	(0.04)
Congruence	0.17^{***}	0.16^{*}
	(0.04)	(0.07)
Item truth		0.87^{***}
		(0.12)
Ideology * Congruence	-0.16^{***}	-0.07
	(0.04)	(0.06)
Ideology * Truth		-0.07
		(0.04)
Congruence * Truth		-0.03
		(0.10)
Ideology * Congruence * Truth		0.01
		(0.08)
Constant	3.17^{***}	2.61^{***}
	(0.07)	(0.11)
Observations	10,431	10,431
R^2	0.01	0.07
Note:	$^{*}p<0.05$; $^{**}p<0.01$; $^{***}p<0.001$	

I.2 Favorability instead of consistence

Table 14 show results for RQ2 and RQ3 using a the "favorability" instead of "consistence" measure of ideological valuence

Table 14: Bias: congruence with favorable instead of consistent pre-test

	Belief	
	RQ2	RQ3
Ideology	0.05	0.07
	(0.04)	(0.06)
Congruence	0.18**	0.25
	(0.06)	(0.13)
Item truth		0.93***
		(0.13)
Ideology * Congruence	−0.17**	−0.13
	(0.06)	(0.10)
Ideology * Truth		−0.11
		(0.06)
Congruence * Truth		−0.17
		(0.14)
Ideology * Congruence * Truth		0.10
		(0.12)
Constant	3.14***	2.55***
	(0.08)	(0.12)
Observations	7,234	7,234
R^2	0.01	0.06
Note:	*p<0.05; **p<0.01; ***p<0.001	

I.3 Including subjects who did not finish

Tables 15 shows results when those who did not finish the questionnaire are included, which gives a sample size of n = 1641.

Table 15: Truth discernment and bias: Including dropouts

	Belief		
	RQ1	RQ2	RQ3
Ideology	0.03	−0.04*	−0.01
	(0.03)	(0.02)	(0.04)
Item truth	0.89***		0.85***
	(0.10)		(0.11)
Ideology * Truth	−0.09**		−0.06
	(0.03)		(0.04)
Truth * Congruence			−0.02
			(0.10)
Ideology * Truth * Congruence			0.01
			(0.07)
Congruence		0.16***	0.15*
		(0.04)	(0.07)
Ideology * Congruence		−0.15***	−0.06
		(0.04)	(0.05)
Constant	2.57***	3.17***	2.62***
	(0.09)	(0.06)	(0.10)
Observations	11,075	11,075	11,075
R^2	0.06	0.01	0.06
Note:	*p<0.05; **p<0.01; ***p<0.001		

I.4 Partisanship instead of ideology

Table 16 shows results for RQ1, RQ2 and RQ3 using partisanship instead of ideology as the underlying political predisposition.

Table 16: Truth discernment and bias: With partisanship

	Belief		
	RQ1	RQ2	RQ3
Partisanship	0.07	0.08	0.33***
	(0.07)	(0.08)	(0.10)
Report truth	0.88***		0.94***
	(0.11)		(0.10)
Partisanship * Truth	−0.18*		−0.45***
	(0.07)		(0.11)
Congruence * Truth			−0.17
			(0.13)
Partisanship * Congruence * Truth			0.54**
			(0.18)
Congruence		0.13*	0.24*
		(0.06)	(0.11)
Partisanship * Congruence		−0.30*	−0.55***
		(0.12)	(0.15)
Constant	2.56***	3.13***	2.55***
	(0.09)	(0.08)	(0.08)
Observations	11,125	7,234	7,234
R^2	0.06	0.01	0.07
Note:	*p<0.05; **p<0.01; ***p<0.001		
